# High fructose consumption aggravates inflammation by promoting effector T cell generation via inducing metabolic reprogramming

**DOI:** 10.1038/s41392-025-02359-9

**Published:** 2025-08-26

**Authors:** Xiao Ma, Jiao Chen, Fang Wang, Xinzou Fan, Zhenhong Li, Hantian Liang, Hao Cheng, Fang Nan, Yubin Lin, Xiaoshuang Song, Jianan Zhang, Fan Gao, Wei Zhang, Wenwen Jin, Huiyuan Zhang, Jiyu Tong, Hong Jiang, Xikun Zhou, Qiang Zou, Hongbo Hu, Aiping Tong, WanJun Chen, Dunfang Zhang

**Affiliations:** 1 Department of Biotherapy, State Key Laboratory of Biotherapy and Cancer Center, Collaborative Innovation Center of Biotherapy, West China Hospital, Sichuan University, Chengdu, Sichuan China; 2 Center for Immunology and Hematology, Department of Biotherapy and Cancer Center and State Key Laboratory of Biotherapy, West China Hospital, Sichuan University, Chengdu, Sichuan China; 3 State Key Laboratory of Oral Diseases and National Center for Stomatology and National Clinical Research Center for Oral Diseases, West China Hospital of Stomatology, Sichuan University, Chengdu, Sichuan China; 4 Innovation Center of Nursing Research, Nursing Key Laboratory of Sichuan Province, West China Hospital, Sichuan University, Chengdu, Sichuan China; 5 Mucosal Immunology Section, National Institute of Dental and Craniofacial Research, National Institutes of Health, Bethesda, MD USA; 6 Department of Immunology, West China School of Basic Medical Sciences and Forensic Medicine, Key Laboratory of Birth Defects and Related Diseases of Women and Children, West China Second University Hospital, Sichuan University, Chengdu, Sichuan China; 7 Department of Pancreatic Surgery, State Key Laboratory of Biotherapy and Cancer Center, West China Hospital, Sichuan University, Chengdu, Sichuan China; 8 Shanghai Chest Hospital & Shanghai Institute of Immunology, State Key Laboratory of Systems Medicine for Cancer, Shanghai Jiao Tong University School of Medicine, Shanghai, China

**Keywords:** Adaptive immunity, Inflammation, Adaptive immunity

## Abstract

The intake of sugars, especially glucose and fructose, has significantly increased with the change of lifestyle. Excessive intake of sugar has been proven to be associated with tumors and inflammatory diseases. Fructose directly mediates innate immune responses; however, whether it can directly regulate T-cell immunity remains unknown. We show that high fructose consumption accelerates the development of inflammatory bowel disease (IBD) by promoting the generation of T helper 1 (Th1) and T helper 17 (Th17) cells. It was demonstrated that fructose promotes the differentiation of Th1 and Th17 cells directly by enhancing mechanistic target of rapamycin complex 1 (mTORC1) activation through the glutamine metabolism-dependent pathway. Reactive oxygen species (ROS)-induced activation of transforming growth factor-β (TGF-β) is also involved in fructose-induced Th17 cell generation. Moreover, metformin can reverse Th1 and Th17 cell generation induced by fructose by suppressing mTORC1 activation and reducing ROS-mediated TGF-β activation. Finally, we identified metformin as an in vivo therapeutic drug for relieving high fructose consumption-induced T-cell inflammation and colitis aggravation. Our study revealed a previously unknown adverse effect of high fructose consumption in disrupting immune homeostasis and exacerbating IBD by directly promoting T-cell immunity, and showed metformin is a potential therapeutic for reversing the T cell immune imbalance caused by long-term high fructose consumption.

## Introduction

Sucrose and high-fructose corn syrup (HFCS) are dietary sugars most commonly added to food, leading to substantial increases in human glucose and fructose intake. The prevalence of high sugar intake is associated with the onset or progression of many diseases, including hyperglycemia, type 2 diabetes (T2D),^1^ tumors,^2^ and cardiovascular diseases.^3^ High fructose intake is a well-known major cause of metabolic dysfunction-associated steatohepatitis (MASH),^4^ colon cancer, and other metabolic diseases.^5^ In recent years, a large amount of epidemiological evidence has shown that the excessive intake of sugary beverages rich in fructose is significantly associated with an increased risk of pancreatic cancer, colorectal cancer, endometrial cancer, and ovarian cancer, and is associated with poor prognosis in breast cancer patients.^6^ Research has gradually elucidated the regulatory mechanisms underlying the impacts of high fructose consumption on tumors. In some tumors, such as lung cancer, acute myeloid leukemia, and prostate cancer, tumor cells can up-regulate the expression of the major fructose transporter GLUT5 (encoded by the *Slc2a5* gene), thereby enhancing their ability to uptake and utilize fructose.^7–9^ Recently, high fructose levels were shown to enhance the acylation of O-linked β-N-acetylglucosamine (O-GlcNAc) through the acetic acid produced by the intestinal flora, promoting the occurrence of hepatocellular carcinoma.^10^ Another research reported high fructose can promote intestinal tumor development by enhancing intestinal cell survival and increasing nutrient absorption.^2^ However, its impact on the immune system has not received sufficient attention.

Colitis incidence has increased significantly worldwide over the past years.^11^ Changes in eating habits are considered one of the main reasons for the increased incidence.^11^ Our previous study demonstrated that high glucose intake can worsen colitis by promoting the activation of transforming growth factor-β (TGF-β) and the differentiation of T helper-17 (Th17) cells.^12^ Another study showed that dietary sugars, including glucose, fructose, and sucrose, can aggravate colitis by altering the microbial population.^11^ Jones et al. found that fructose can promote the production of interleukin-1β (IL-1β) in lipopolysaccharide (LPS)-stimulated monocytes and macrophages through cellular metabolic reprogramming.^13^ However, another study reported that fructose suppresses M1-like tumor-associated macrophages and promotes colorectal cancer growth,^14^ showing that the impacts of fructose on macrophages should be closely related to the specific tissue microenvironment. Recently, Zhang et al. reported that high fructose consumption increases the activation of the mechanistic target of rapamycin complex 1 (mTORC1) to upregulate leptin expression in adipocytes by promoting the accumulation of glycolytic intermediates, thereby contributing to CD8^+^ T cell-mediated anti-tumor immune response in tumor-bearing mice.^15^ These findings show that fructose can indirectly affect T cell immunity by influencing adipose tissue. Although fructose has been found to be able to regulate innate immune responses by inducing metabolic reprogramming in monocytes and macrophages and regulate CD8^+^ T cell immunity indirectly by promoting leptin expression of adipose tissue, whether it can directly regulate acquired immunity, especially T-cell immunity, still lacks sufficient research.

In tumor cells, macrophages, and adipocytes, fructose-induced metabolic reprogramming has all led to the activation of the mechanistic target of rapamycin (mTOR) signaling pathway.^9,13,15^ The mTOR pathway can integrate various signals in the immune microenvironment and regulate multiple functions of immune cells, including immune cell growth, apoptosis, and differentiation.^16,17^ It has been shown that when the mTOR signaling pathway in CD4^+^ T cells is defective, CD4^+^ T cells cannot differentiate into effector T cells even under appropriate induction conditions.^18^ At present, there is still a lack of systematic and in-depth research on the impact of fructose on T cell metabolism, and it is even unclear whether T cells can effectively take up and utilize fructose. Therefore, whether fructose can regulate the differentiation and function of T cells through the mTOR signaling pathway has not yet been revealed. Recently, a study showed that T cells lack the expression of the fructose transporter GLUT5, suggesting that T cells may not be able to effectively utilize fructose.^19^ Notably, fructose metabolism was reported to upregulate glutamine metabolism in macrophages.^13^ Moreover, glutamine metabolism has been demonstrated to be able to promote the activation of the mTOR signaling pathway in different cell types.^20,21^ Therefore, whether fructose can activate the mTOR signaling pathway in T cells to directly regulate effector T cell differentiation and function by upregulating glutamine metabolism is of great research significance.

Here, we revealed that high fructose consumption exacerbates dextran sulfate sodium salt (DSS)-induced colitis and CD4^+^CD25^−^CD45RB^hi^ T cell transfer-induced colitis by promoting the generation of T helper-1 (Th1) and Th17 cells. We further demonstrate that fructose promotes the differentiation of Th1 and Th17 cells by upregulating the mTORC1 through the glutamine metabolism-dependent pathway. Reactive oxygen species (ROS)-induced activation of latent TGF-β is also involved in fructose-induced Th17 cell generation. Based on these findings, we further demonstrate that metformin reverses fructose-induced Th1 and Th17 cell generation by suppressing mTORC1 activation. Metformin can also suppress Th17 cell generation by reducing ROS-mediated TGF-β activation. These findings highlight a previously unidentified adverse effect of high fructose consumption in promoting T cell inflammation that can be reversed by metformin, a promising therapeutic for clinical applications.

## Results

### High fructose consumption promotes Th1 and Th17 cell-mediated immunity

**Fig. 1:**
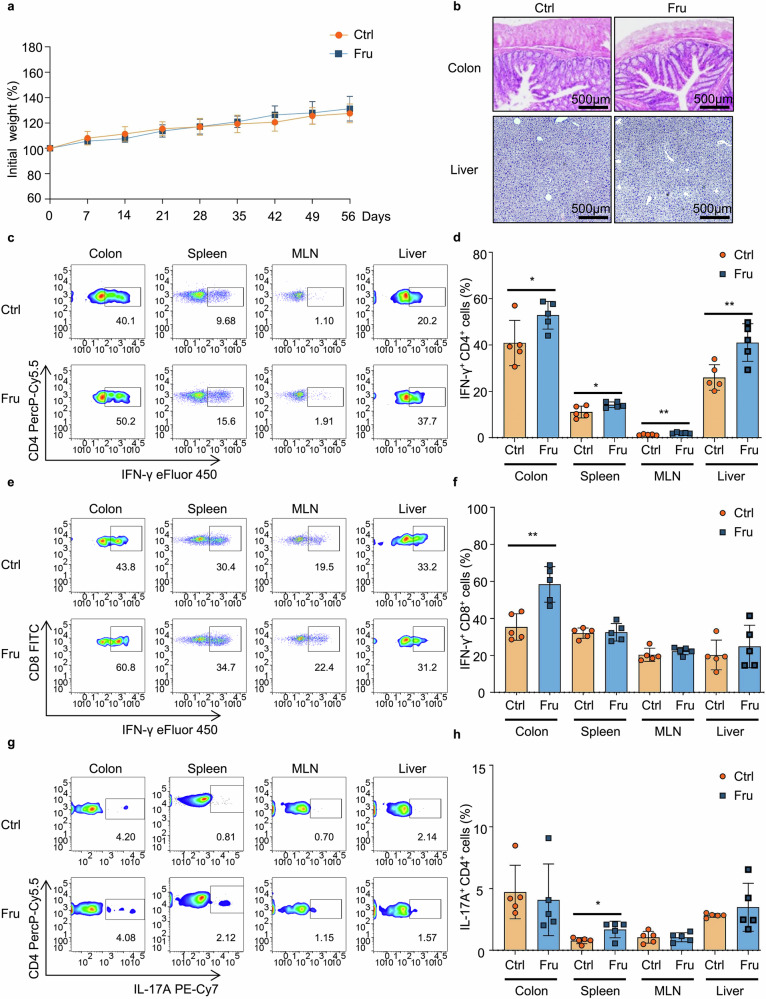
High fructose consumption promotes Th1 and Th17 cell-mediated immunity. T cell immune responses in C57BL/6 mice treated with 20% fructose water for 2 months were investigated (*n* = 5). One of two independent experiments was showed. **a** Body weight changes in control- (Ctrl) and fructose (Fru)-treated mice. **b** H&E-stained colon and liver sections. (**c–h**) Frequencies of IFN-γ^+^CD4^+^ T (Th1) (**c**, **d**), IFN-γ^+^CD8^+^ T (Tc1) (**e**, **f**), and IL-17A^+^CD4^+^ T (Th17) (**g**, **h**) cells in indicated tissues. Unpaired two-tailed Student’s *t*-tests were used to calculate statistical significance. Data are presented as mean ± SD. *p < 0.05; **p < 0.01

To determine the impacts of high fructose intake on T-cell immune homeostasis, we first examined the changes of blood glucose levels in mice treated with high fructose water. We found that there was no significant change in blood sugar levels in mice fed with fructose (Fru group) compared to control mice (Ctrl group) (Supplementary Fig. 1a). In contrast, the fructose content in the serum of high fructose water-treated mice was significantly increased (Supplementary Fig. 1b). There were no significant differences in diet or water intake between control mice and mice fed with high fructose water (Supplementary Fig. 1c). The body weight and histopathology of the control mice and mice fed with high fructose water showed no significant changes (Fig. 1a, b), which was consistent with a previous finding that feeding mice with high fructose water did not lead to weight gain.^22^ After 2 months of high fructose feeding, it was determined that the frequency of IFN-γ^+^CD4^+^ T (Th1) cells was upregulated in the colon, spleen, mesenteric lymph nodes (MLN), and liver of high fructose-treated mice (Fig. 1c, d). The frequency of IFN-γ^+^CD8^+^ T (Tc1) cells was also significantly increased in colons of high fructose-treated mice (Fig. 1e, f). The frequency of IL-17A^+^CD4^+^ T (Th17) cells was significantly increased in the spleens of mice fed with high fructose water (Fig. 1g, h). In contrast, the frequencies of CD4^+^IL-10^+^ T (Tr1), CD4^+^IL-4^+^ T (Th2), and CD4^+^Foxp3^+^ regulatory T (Treg) cells did not change significantly (Supplementary Fig. 1d–i). These data demonstrate that high fructose intake can promote the production of IFN-γ and IL-17A in T cells.

### High fructose consumption aggravates inflammatory bowel disease by promoting Th1 and Th17 cell-mediated immunity

**Fig. 2:**
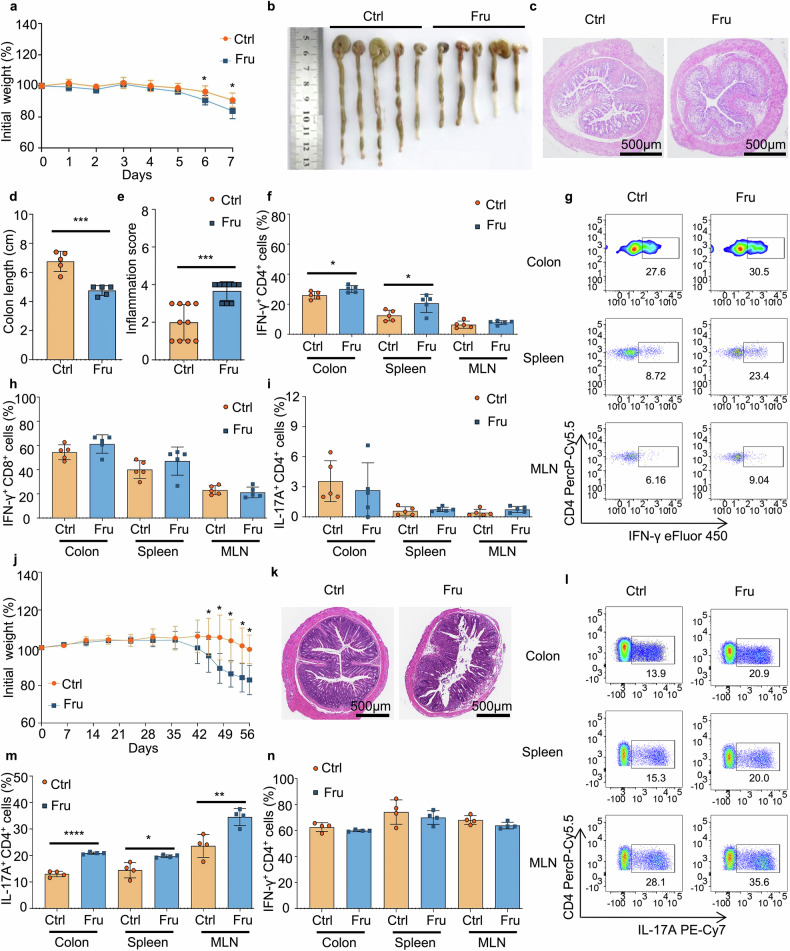
High fructose consumption exacerbates inflammatory bowel disease by enhancing Th1 and Th17 cell-mediated immunity in two disease models. **a–i** The DSS-induced colitis model (*n* = 5). One of two independent experiments was showed. **a** Changes in body weight during the colitis induction. **b** The photograph of the colons. **c** H&E-stained colon sections. **d** The length of the colon. **e** The inflammation score of colitis (*n* = 10, pooled from two independent experiments). **f–i** Frequencies of Th1 (**f**, **g**), Tc1 (**h**), and Th17 (**i)** cells in indicated tissues. **j–n** The T cell transfer colitis model (*n* = 4). One of two independent experiments was showed. **j** Changes in body weight during colitis development. **k** H&E-stained colon sections. Frequencies of Th17 cells (**l**, **m)** and Th1 cells (**n)** in the colon, spleen, and MLN. Unpaired two-tailed Student’s *t* tests were used to calculate statistical significance. Data are presented as mean ± SD. *p < 0.05; **p < 0.01; ****p < 0.0001

The finding that high fructose consumption promoted Th1 and Th17 cell generation encouraged us to explore whether high fructose consumption plays a role in colonic inflammation. First, we established a DSS-induced colitis model in mice treated with high fructose or normal drinking water. The mice in the Fru group lost more weight than the mice in the control group (Fig. 2a). To verify the severity of colitis, we collected the colons of these mice and analyzed their length and inflammation after euthanization. The colons in the Fru group were relatively shorter, and inflammation was significantly more severe in the Fru group than in the control group (Fig. 2b–e). We then investigated the immune responses using flow cytometry, finding that the frequency of Th1 cells significantly increased in the colon and spleen of mice treated with high fructose water (Fig. 2f, g). In contrast, the Tc1, Th17, Tr1, Th2, and Treg cell frequencies in the colon did not differ significantly between Ctrl and Fru groups (Fig. 2h, i and Supplementary Fig. 2a–c). These data show that high fructose consumption promotes Th1 cell generation in this DSS-induced colitis model.

The intestine, the largest immune organ, is rich in IL-6.^23^ Th17 cell immunity plays an essential role in inflammatory bowel disease. However, no significant Th17 cell immune response was observed in the DSS-induced colitis model (Fig. 2i). To explore whether high fructose consumption regulates the Th17 cell immune response in inflammatory bowel disease, we established a T cell-transfer colitis model in which Th1 and Th17 cells are involved in disease development. *Rag1*^*−/−*^ mice were randomly divided into control and Fru groups, and high fructose treatment was initiated 2 days before CD4^+^CD25^-^CD45RB^hi^ naïve T cell transfer. Mice in the Fru group showed more severe weight loss and pathological changes than mice in the control group (Fig. 2j, k). We found that the frequency of Th17 cells was significantly increased in the colon, spleen, and MLN of mice fed high fructose water in this model (Fig. 2l, m). Owing to the high frequency of Th1 cells in this T cell-transfer colitis model, we did not observe an increase in Th1 cells in mice fed high fructose water (Fig. 2n, Supplementary Fig. 2d). Tr1, Th2, and Treg cell frequencies did not change significantly in the colon (Supplementary Fig. 2e–g). Taken together, high fructose consumption promotes Th17 cell generation in this T cell-transfer colitis model.

To investigate whether the exacerbation of colitis induced by high fructose consumption was caused by enhanced T cell-mediated inflammation, we established DSS-induced colitis in nude mice with T cell deficiency. No significant differences were observed in colitis disease severity or weight loss between the high fructose and normal drinking water-treated mice (Supplementary Fig. 2h, i). Moreover, *Rag1*^−/−^ mice were also used to establish the DSS-induced colitis model, and the results were consistent with those of the nude mice (Data not shown). Therefore, high fructose supplementation did not exacerbate DSS-induced colitis without T cells. These data demonstrate that high fructose consumption in drinking water exacerbates IBD progression by promoting the generation of Th1 and Th17 cells.

### Fructose promotes Th1 and Th17 cell differentiation directly in vitro

**Fig. 3:**
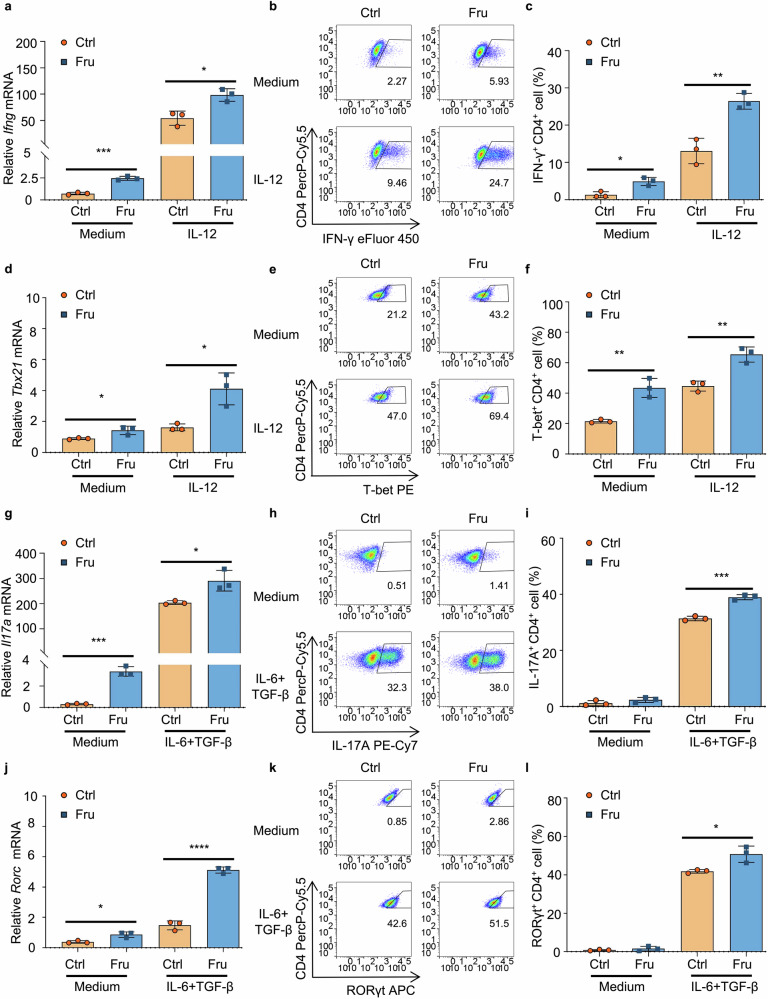
Fructose directly promotes differentiation of Th1 and Th17 cells in vitro. Naïve T cells were cultured in cDMEM containing 25 mM glucose or 25 mM fructose in the presence of indicated cytokines (*n* = 3). Th1 cells were induced with recombinant mouse IL-12 (10 ng/mL), and Th17 cells were induced with recombinant human TGF-β1 (2 ng/mL) and recombinant mouse IL-6 (50 ng/mL). Cells were cultured at 37 °C, 5% CO_2_ for 3 days. **a–c** The RNA level of *Ifng* (**a)** and Th1 cell frequencies (**b**, **c)** under indicated cell culture conditions. **d–f** The RNA level of *Tbx21* (**d)** and T-bet^+^CD4^+^ T cell frequencies (**e**, **f)** under indicated cell culture conditions. **g–i** The RNA level of *Il17a* (**g)** and Th17 cell frequencies (**h**, **i)** under indicated cell culture conditions. **j–l** The RNA level of *Rorc* (**j)** and RORγt^+^CD4^+^ T cell frequencies (**k**, **l)** under indicated cell culture conditions. Unpaired two-tailed Student’s *t* tests were used to calculate statistical significance. Summary data are presented as mean ± SD. *p < 0.05; **p < 0.01; ***p < 0.001; ****p < 0.0001

Although we demonstrated that high fructose consumption can increase the generation of Th1 and Th17 cells in vivo, whether fructose directly regulates Th1 and Th17 cell generation is unknown. CD4^+^CD25^-^CD62L^+^ naïve T cells were cultured with complete DMEM (cDMEM) containing glucose (Ctrl) or fructose (Fru) in Th1 and Th17 cell differentiation conditions to investigate this. It was identified that fructose promoted the expression of IFN-γ and T-bet, the key transcription factor of Th1 cells, at both RNA and protein levels under Th0 and Th1 cell-induction conditions, showing that fructose can promote Th1 cell differentiation (Fig. 3a–f). Fructose also promoted the expression of IL-17A and retinoic acid-related orphan receptor γt (RORγt), the key transcription factor of Th17 cells, at both RNA and protein levels under Th17 cell-induction conditions, showing that fructose can also promote Th17 cell differentiation (Fig. 3g–l). Under Th1 and Th17 cell-inducing conditions, fructose did not affect the differentiation of Treg, Tr1, or Th2 cells (Supplementary Fig. 3a–f). These data demonstrate that fructose directly promotes the differentiation of Th1 and Th17 cells in vitro.

### Th1 and Th17 cell differentiation promoted by fructose is not through the regulation of T cell activation

**Fig. 4:**
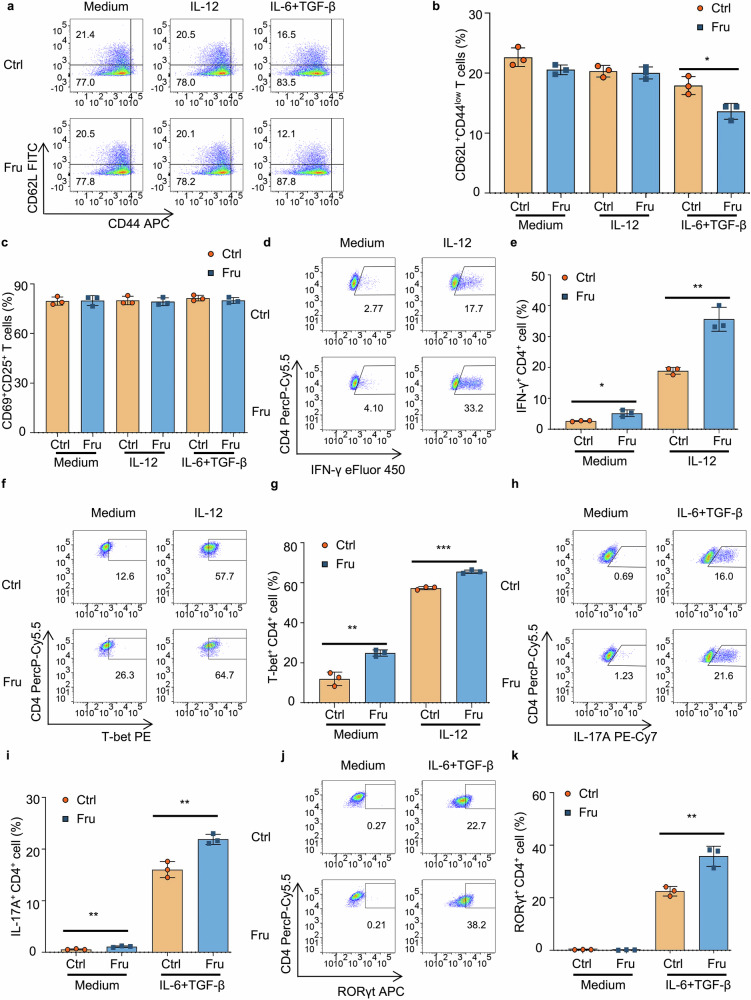
Th1 and Th17 cell differentiation promoted by fructose is not through the regulation of T-cell activation. **a**–**c** Naïve T cells were cultured in cDMEM containing 25 mM glucose or 25 mM fructose in the presence of indicated cytokines for 24 h (*n* = 3). Frequencies of CD62L^+^CD44^low^ inactivated T cells (**a**, **b)** and CD69^+^CD25^+^ activated T cells (**c)** in T cells cultured in Th0, Th1, and Th17 cell induction conditions. **d**–**k** Naïve T cells were cultured in a normal cDMEM for 24 h. TCR stimulation was then removed, and T cells were re-cultured in cDMEM containing 25 mM glucose or 25 mM fructose in the presence of IL-2 (10 ng/mL), with or without IL-12 (for Th1 cell induction) or TGF-β1 plus IL-6 (for Th17 cell induction), for 2 days (*n* = 3). **d**, **e** Th1 cell frequencies under indicated cell culture conditions. **f**, **g** T-bet^+^CD4^+^ T cell frequencies under indicated cell culture conditions. **h**, **i** Th17 cell frequencies under indicated cell culture conditions. **j**, **k** RORγt^+^CD4^+^ T cell frequencies under indicated cell culture conditions. Unpaired two-tailed Student’s *t* tests were used to calculate statistical significance. Data are presented as mean ± SD. *p < 0.05; **p < 0.01; ***p < 0.001

Because the activation of T cells is indispensable for the growth and differentiation of T cells, next the effect of fructose on T cell activation was investigated. We found that fructose did not affect T cell activation-associated markers, including CD62L, CD44, CD69, and CD25, under Th0 and Th1 cell induction conditions (Fig. 4a–c and Supplementary Fig. 4a). However, fructose promoted early activation of T cells by downregulating CD62L faster under Th17 cell induction condition but did not influence the late stage of T cell activation (Fig. 4a–c and Supplementary Fig. 4a). To further explore whether fructose induced Th1 and Th17 cell differentiation is through promoting T cell activation, T cells were cultured in a standard complete medium for 24 h to be activated fully (Fig. 4a–c and Supplementary Fig. 4a, Ctrl sample under Th0 induction condition); then the activated T cells were washed to remove the T cell receptor (TCR) stimulation and re-cultured in Ctrl or Fru medium, with or without IL-12 for Th1 cell induction or IL-6 plus TGF-β for Th17 cell induction. The results showed that fructose still promoted the differentiation of Th1 and Th17 cells (Fig. 4d–k), indicating that the effect of fructose on T-cell differentiation does not rely on fructose-mediated T-cell activation. We also investigated the effects of fructose on T-cell apoptosis and proliferation, finding that fructose had no significant impact on T-cell apoptosis (Supplementary Fig. 4b, c). Fructose did not promote T cell proliferation and actually slightly suppressed T cell proliferation (Supplementary Fig. 4d, e), indicating that the increases of Th1 and Th17 cells induced by fructose are not achieved by promoting cell proliferation. Taken together, these data demonstrate that the differentiation of Th1 and Th17 cells promoted by fructose is not through regulating T cell activation.

### Fructose-induced Th1 and Th17 cell differentiation is dependent on glutamine metabolism

**Fig. 5:**
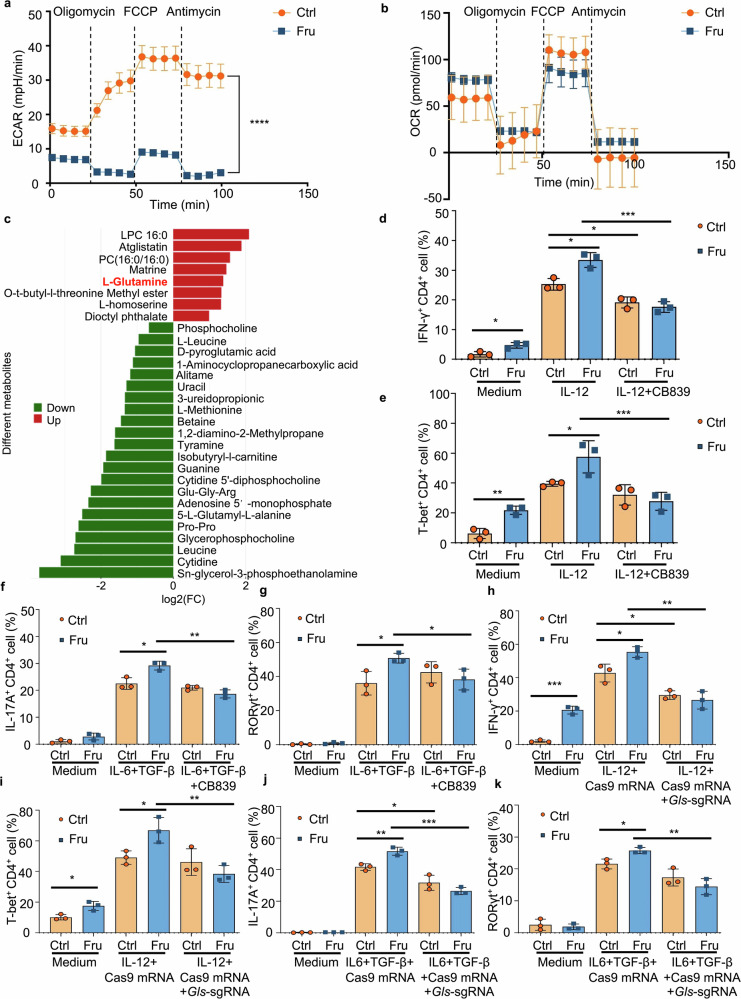
Fructose promotes Th1 and Th17 cell differentiations through a glutamine metabolism-dependent pathway. **a**, **b** The ECAR and OCR of CD4^+^ T cells incubated in glucose or fructose medium for 24 h were determined (*n* = 3). **c** Analysis of the significantly different metabolites in T cells cultured in cDMEM containing fructose or glucose for 3 days (the horizontal coordinate in the Figure represents the log2 FC value of the differential metabolites, and the vertical coordinate represents the significantly different metabolites. Red shows upregulated differential metabolites in fructose-treated T cells, and blue shows downregulated differential metabolites in fructose-treated T cells. **d**–**k** Naïve T cells were cultured in cDMEM containing 25 mM glucose or fructose, with or without indicated cytokines and reagents. Glutamine metabolism pathway was blocked with a glutaminase inhibitor CB839 (1 μM) or by *Gls* gene mutation using CRISPR/CAS9 system. Cells were cultured at 37 °C, 5% CO_2_ for 3 days (*n* = 3). Frequencies of IFN-γ^+^ CD4^+^ Th1 cells (**d**), T-bet^+^ CD4^+^ T cells (**e**), IL-17A^+^ CD4^+^ Th17 cells (**f**), and RORγt^+^ CD4^+^ T cells (**g**) under indicated cell culture conditions. Frequencies of IFN-γ^+^ CD4^+^ Th1 cells (**h**), T-bet^+^ CD4^+^ T cells (**i**), IL-17A^+^ CD4^+^ Th17 cells (**j**), and RORγt^+^ CD4^+^ T cells (**k**) in control T cells or *Gls* mutant T cells under indicated cell culture conditions. Unpaired two-tailed Student’s *t* tests were used to calculate statistical significance. Data are presented as mean ± SD. *p < 0.05; **p < 0.01; ***p < 0.001; ****p < 0.0001

We sequenced bulk RNA (RNA-seq) to investigate how fructose regulates Th1 and Th17 cell differentiation. No significant differences were observed in genes related to T cell activation or differentiation between the fructose and glucose groups under Th0, Th1, or Th17 culture conditions (Supplementary Fig. 5a).^13,24,25^ Metabolic pathways are demonstrated to play vital roles in regulating T cell responses;^26^ we expected that fructose-induced metabolic reprogramming would affect Th1 and Th17 cell differentiation. Since glycolysis is the primary metabolic pathway of hexoses, including glucose and fructose,^27^ we determined the level of T-cell glycolysis using Seahorse real-time cell metabolic analyses. The Extracellular Acidification Rate (ECAR) of fructose-cultured T cells was dramatically lower than that of the control T cells, indicating that glycolysis was largely suppressed in fructose-treated T cells (Fig. 5a). In the meanwhile, the Oxygen Consumption Rate (OCR) of fructose medium-cultured T cells was comparable to that of control medium-cultured T cells (Fig. 5b), indicating that fructose inhibited T cell glycolysis but did not affect mitochondrial metabolism. We also analyzed the levels of basal glycolysis, glycolytic capacity, and maximal respiration in T cells cultured in control and Fru media. The results showed that basal glycolysis and glycolytic capacity levels were significantly suppressed in T cells cultured in Fru medium (Supplementary Fig. 5b, c). However, the level of maximal respiration showed no significant difference between the two groups (Supplementary Fig. 5d). RNA-seq data and Q-PCR results showed that T cells do not express *Slc2a5*, the gene encodes for fructose transporter GLUT5 (Supplementary Fig. 5e, f), showing that T cells cannot transport and utilize fructose effectively. These data suggest that other metabolic pathways may be activated to compensate for the effect of reduced glycolysis on mitochondrial metabolism. Previous studies have found that, in addition to glycolysis, several metabolic pathways, including glutamine metabolism, are involved in T-cell activation and function.^25,28,29^ Therefore, we conducted a non-target metabolomic analysis to determine whether fructose can induce metabolic reprogramming in T cells in addition to suppressing glycolysis (Supplementary Fig. 5g). We found that glutamine levels increased in fructose-treated T cells (Fig. 5c). Therefore, we hypothesized that glutamine metabolism is up-regulated in the response to fructose-induced reduction of T-cell glycolysis and is essential for fructose-mediated T-cell differentiation. To investigate whether glutamine metabolism is key to fructose-induced Th1 and Th17 cell differentiation, T cells were cultured with a glutaminase inhibitor CB-839 under Th1 and Th17 cell culture conditions. Surprisingly, the results showed that CB-839 reversed the increase of Th1 cell differentiation induced by fructose at both the RNA and protein levels (Fig. 5d, e and Supplementary Fig. 6a–c). CB-839 also reversed the increase of Th17 cell differentiation induced by fructose at both the RNA and protein levels (Fig. 5f, g and Supplementary Fig. 6d–f). The differentiation of Th1 and Th17 cells in the control medium was not affected dramatically by CB-839 (Fig. 5d–g and Supplementary Fig. 6a–f). To further confirm the role of glutamine metabolism in fructose-induced Th1 and Th17 cell differentiation, *Gls* gene was deleted in T cells using CRISPR/CAS9 system. The results showed that fructose-induced generation of Th1 and Th17 cells was also reversed in glutaminase-deficient T cells (Fig. 5h–k and Supplementary Fig. 6g, h). These data indicated that fructose-induced Th1 and Th17 cell differentiation is dependent on glutamine metabolism.

### Fructose promotes Th1 and Th17 cell differentiation via glutamine metabolism-dependent mTORC1 activation

**Fig. 6:**
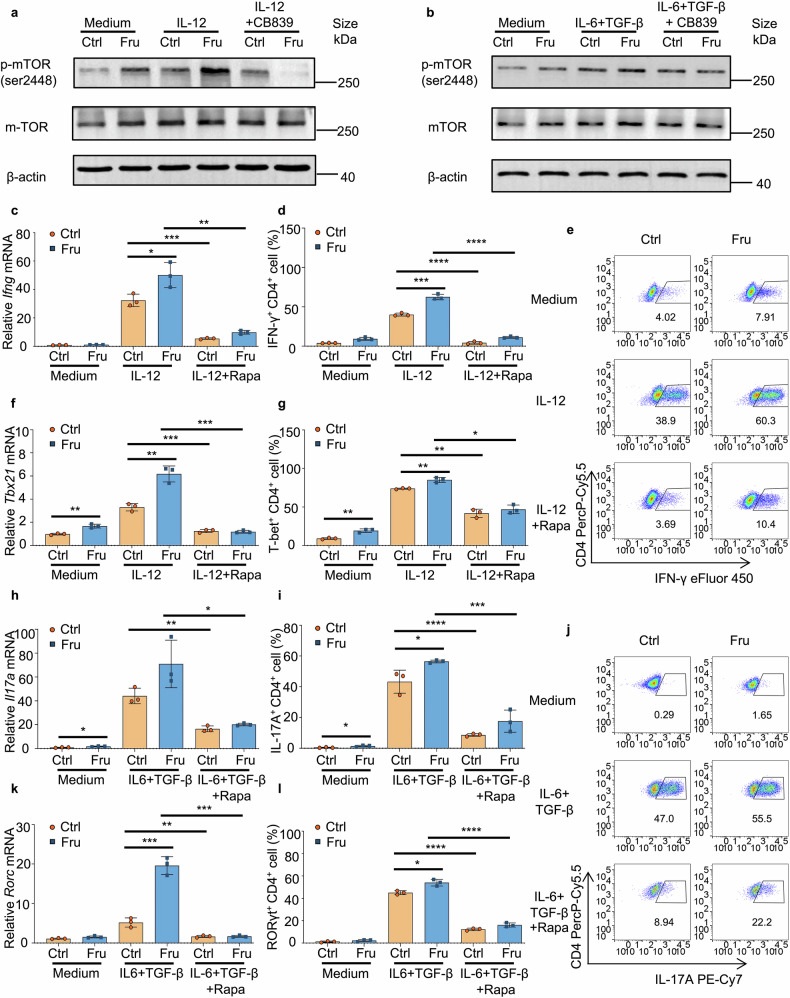
Fructose promotes differentiation of Th1 and Th17 cells via glutamine metabolism-dependent mTORC1 activation. Naïve T cells were cultured in glucose medium or fructose medium for 24 h or 3 days, with or without indicated cytokines and reagents (*n* = 3). **a**, **b** Western blot analysis of mTOR and phospho-mTOR (Ser2448) in T cells post 24 h culture. **c–e** The RNA level of *Ifng* (**c)** and frequencies of Th1 cells (**d**, **e)** in T cells post 3 days culture. **f, g** The RNA level of *Tbx21* (**f)** and frequencies of T-bet^+^CD4^+^ T cells (**g)** in T cells post 3 days culture. **h–j** The RNA level of *Il17a* (**h)** and frequencies of Th17 cells (**i**, **j)** in T cells post 3 days culture. **k, l** The RNA level of *Rorc* (**k)** and frequencies of RORγt^+^CD4^+^ T cells (**l)** in T cells post 3 days culture. Unpaired two-tailed Student’s *t* tests were used to calculate statistical significance. Data are presented as mean ± SD. *p < 0.05; **p < 0.01; ***p < 0.001; ****p < 0.0001

We next investigated the molecular mechanism by which fructose promotes Th1 and Th17 cell differentiation. It has been shown that the increase in glutamine metabolism supports the activation of mTORC1 in the presence of fructose and promotes the expression of downstream inflammatory factors.^13,30^ Jiang et al. reported that mTOR signaling promotes periodontitis progression by promoting the differentiation of Th1 and Th17 cells.^31^ To identify the activation of mTORC1 in fructose-treated T cells, T cells were cultured under Th0, Th1, and Th17 cell induction conditions, and western blotting was performed to determine the levels of phospho-mTOR (Ser2448). The levels of phospho-mTOR (Ser2448) were significantly up-regulated in fructose-treated T cells, and the administration of CB-839 reversed phosphorylation of mTOR (Ser2448) in fructose-treated but not in glucose-treated T cells (Fig. 6a, b), indicating that fructose-induced phosphorylation of mTOR (Ser2448) is highly dependent on glutamine metabolism. To further explore the function of mTORC1 activation in fructose-induced T cell differentiation, we cultured T cells with rapamycin, a mTORC1 inhibitor, under Th1 and Th17 cell induction conditions in control or Fru medium. The results showed that rapamycin significantly suppressed the increase of IFN-γ-producing Th1 cell generation and T-bet expression induced by fructose at both the RNA and protein levels (Fig. 6c–g). Rapamycin also suppressed the increase of IL-17A-producing Th17-cell generation and transcription factor RORγt expression induced by fructose at both the RNA and protein levels (Fig. 6h–l). Besides, rapamycin also suppressed the generation of Th1 and Th17 cells in a glucose-containing medium (Fig. 6c–l), showing that mTORC1 is important for effector T-cell differentiation. In contrast, rapamycin treatment did not affect Th2 or Tr1 cells under Th1 and Th17 cell induction conditions and only slightly suppressed Treg differentiation under Th17 cell induction conditions (Supplementary Fig. 7a–h). Based on these data, we conclude that fructose promotes the differentiation of Th1 and Th17 cells through glutamine metabolism-dependent mTORC1 activation.

### High fructose-induced TGF-β activation by driving ROS production is involved in fructose-induced Th17 cell differentiation

**Fig. 7:**
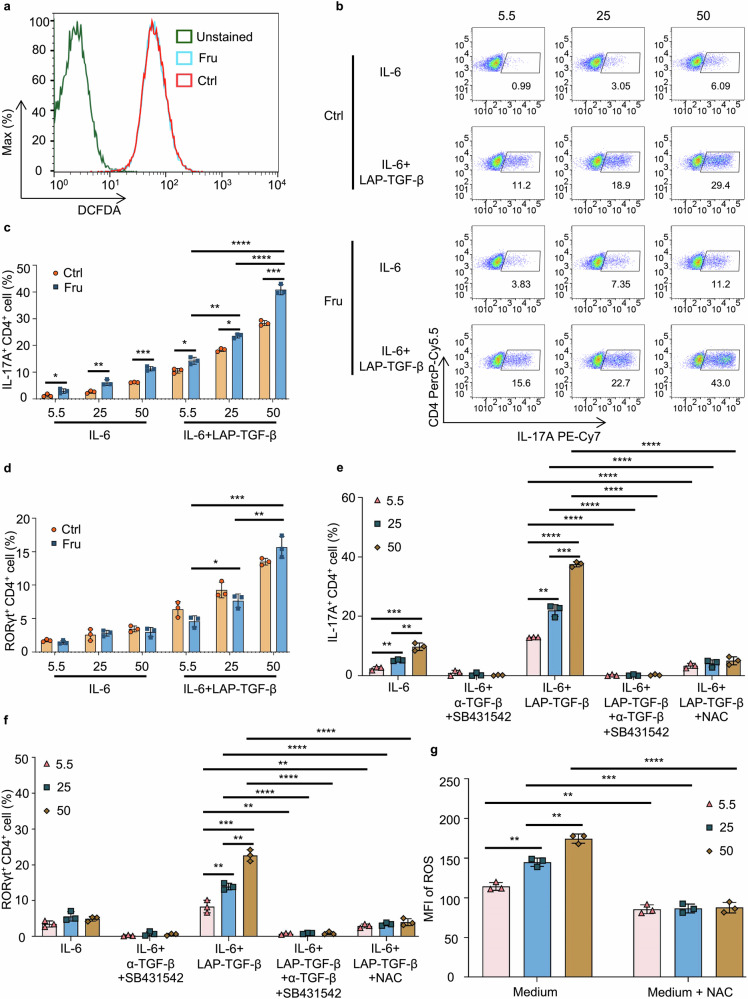
High fructose-induced TGF-β activation is involved in fructose-induced Th17 cell differentiation. **a** Naïve T cells were cultured in glucose medium or fructose medium for 24 h, then the production of ROS was determined (*n* = 3). **b**–**d** Naïve T cells were cultured in cDMEM containing indicated concentrations of glucose or fructose for 3 days (*n* = 3). **b**, **c** Frequencies of Th17 cells in T cells post 3 days culture. **d** Frequencies of RORγt^+^CD4^+^ T cells in T cells post 3 days culture under indicated cell culture conditions. Naïve T cells were cultured in glucose medium or fructose medium for 3 days, with or without indicated cytokines and reagents (*n* = 3). **e, f** Frequencies of Th17 cells (**e)** and RORγt^+^CD4^+^ T cells (**f)** in T cells cultured under indicated cell culture conditions. **g** Naïve T cells were cultured in indicated media for 24 h, then the production of ROS was determined (*n* = 3). Unpaired two-tailed Student’s *t* tests were used to calculate statistical significance. Data are presented as mean ± SD. *p < 0.05; **p < 0.01; ***p < 0.001; ****p < 0.0001

Our previous study proved that high glucose drives ROS production in activated CD4^+^ T cells and promotes the differentiation of Th17 cells through ROS-mediated activation of latent TGF-β.^12^ We determined that fructose and glucose can induce the same level of ROS production (Fig. 7a), so we speculated that fructose might also activate TGF-β by inducing ROS production. To investigate this point, naïve T cells were cultured in complete DMEM containing different concentrations of glucose or fructose, in the presence of latent TGF-β1 and IL-6.^12,32^ The results showed that higher fructose concentrations induced more Th17 cell differentiation (Fig. 7b–d), demonstrating that high fructose can induce Th17 differentiation by promoting the activation of latent TGF-β. The data showed that fructose always induced more Th17 and Th1 cells than glucose at the indicated sugar doses in these cultures (Fig. 7b, c and Supplementary Fig. 8a–c), showing that fructose-induced mTORC1 activation also plays a part in these cell culture conditions. However, these conditions did not significantly affect the differentiation of Th2, Tr1, or Treg cells (Supplementary Fig. 8d–f).

Next, to verify that high fructose can indeed activate TGF-β, T cells were cultured at indicated doses of fructose, in the presence or absence of IL-6, latent TGF-β1, αTGF-β, and SB431542. The results showed that blocking TGF-β signaling pathway with αTGF-β and SB431542 completely inhibited high fructose-induced Th17 cell differentiation in T cells cultured with IL-6 and latent TGF-β1 (Fig. 7e, f). To determine whether higher fructose also increased ROS production, we measured ROS levels in T cells cultured at indicated doses of fructose, with or without the ROS scavenger N-acetyl-L-cysteine (NAC). The results showed that higher fructose levels induced more ROS, and NAC reversed this increase (Fig. 7g and Supplementary Fig. 8g). The results demonstrated that neutralizing ROS with NAC reversed high fructose-induced Th17 cell differentiation in T cells cultured with IL-6 and latent TGF-β1 (Fig. 7e, f). In T cells cultured with IL-6 alone, higher fructose also induced slightly more Th17 cells (Fig. 7e). This is relay on fetal bovine serum (FBS)-contained latent TGF-β1 in complete medium, because blocking TGF-β signaling in this condition also reversed the differentiation of Th17 cells (Fig. 7e, f). These data demonstrated that high fructose-induced TGF-β activation by driving ROS production promotes fructose-induced Th17 cell differentiation.

### Metformin inhibits fructose-induced Th1 and Th17 cell differentiation by suppressing mTORC1

**Fig. 8:**
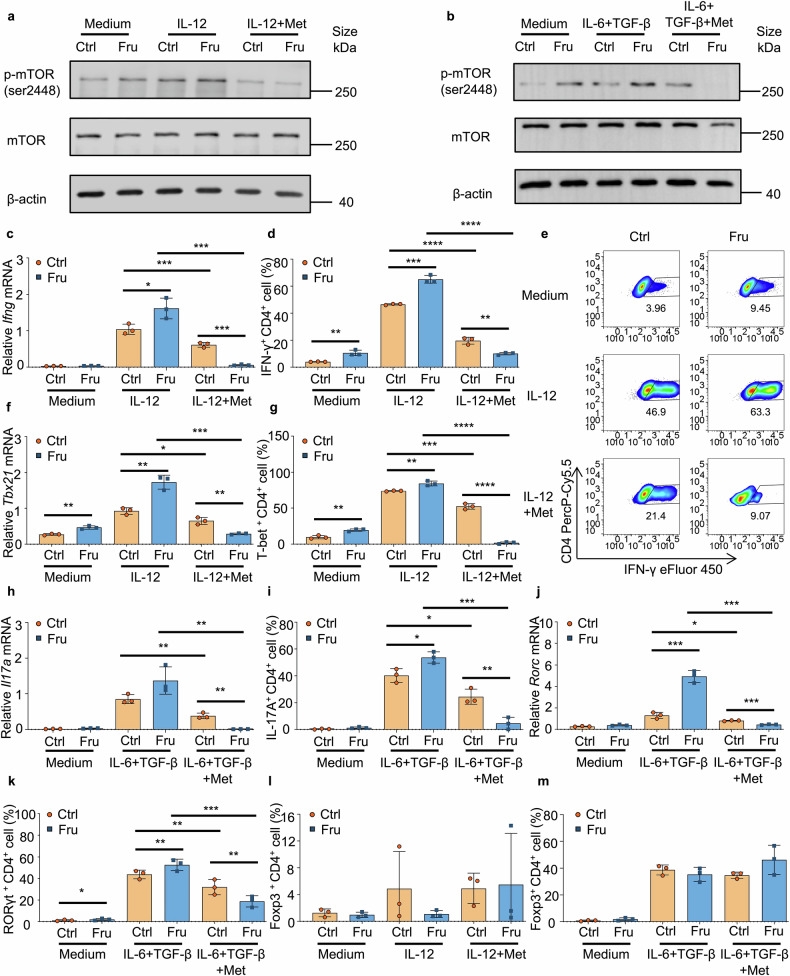
Metformin inhibits fructose-induced Th1 and Th17 cell differentiations by suppressing mTORC1. Naïve T cells were cultured in glucose medium or fructose medium for 24 h or 3 days, with or without indicated cytokines and reagents (*n* = 3). **a**, **b** Western blot analysis of mTOR and phospho-mTOR (Ser2448) in T cells post 24 h culture. **c–e** The RNA level of *Ifng* (**c)** and frequencies of IFN-γ^+^CD4^+^ Th1 cells (**d**, **e)** in T cells post 3 days culture. **f, g** The RNA level of *Tbx21* (**f)** and frequencies of T-bet^+^CD4^+^ T cells (**g)** in T cells post 3 days culture. **h, i** The RNA level of *Il17a* (**h)** and frequencies of Th17 cells (**i)** in T cells post 3 days culture. **j, k** The RNA level of *Rorc* (**j)** and frequencies of RORγt^+^CD4^+^ T cells (**k)** in T cells post 3 days culture. **l**, **m** Frequencies of Foxp3^+^CD4^+^ Treg cells in T cells post 3 days culture. Unpaired two-tailed Student’s *t*-tests were used to calculate statistical significance. Data are presented as mean ± SD. *p < 0.05; **p < 0.01; ***p < 0.001; and ****p < 0.0001

The findings prove that fructose can exacerbate T-cell immunity by promoting Th1 and Th17 cell generation by upregulating mTORC1 and ROS. Next, we investigated whether any drug could reverse fructose-induced T-cell inflammation. Metformin is a well-tolerated and effective drug for treating T2D and improving blood sugar control.^33^ It has been shown that metformin can alleviate intestinal inflammation and repair intestinal barrier structures.^34^ Several studies reported that metformin can inhibit the activation of mTORC1.^35–37^ This prompted us to investigate whether metformin could eliminate fructose-induced Th1 and Th17 cell generation by inhibiting mTORC1. Indeed, metformin reversed fructose-induced phosphorylation of mTOR (Ser2448) in T-cell cultures (Fig. 8a, b). To determine the effect of metformin in fructose-mediated T-cell differentiation, T cells were cultured with metformin under Th1 and Th17 cell induction conditions in a complete medium containing fructose or glucose. The results showed that metformin completely reversed fructose-induced differentiation of Th1 and Th17 cells under indicated induction conditions (Fig. 8c–k). More than that, the generation of Th1 and Th17 cells was inhibited more in fructose-cultured T cells than in control medium-cultured T cells in the presence of metformin (Fig. 8c–k). In contrast, metformin did not significantly affect other T cell subtypes, including Treg, Th2, and Tr1 cells, under Th1 and Th17 cell induction conditions (Fig. 8l, m and Supplementary Fig. 9a–f). Our data show that metformin completely reversed fructose-induced Th1 and Th17 cell generation by suppressing mTORC1 activation.

### Metformin inhibits fructose-induced TGF-β activation by suppressing ROS production

**Fig. 9:**
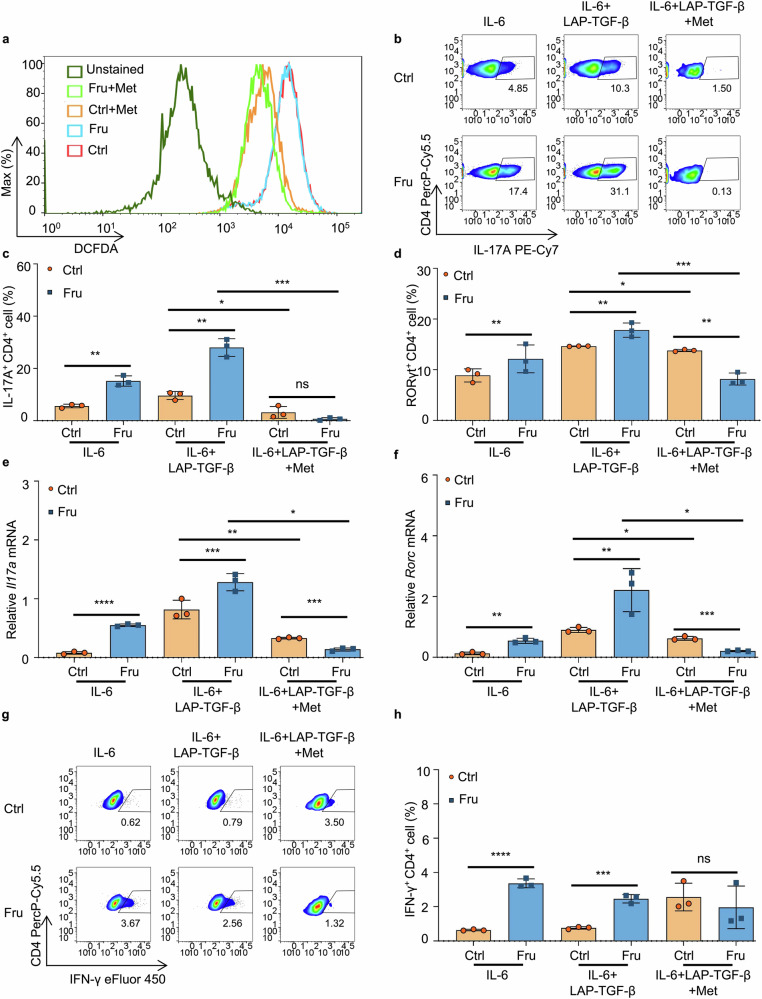
Metformin inhibits fructose-induced TGF-β activation by suppressing ROS production. **a** Naïve T cells were cultured in glucose medium or fructose medium for 24 h, then the production of ROS was determined (*n* = 3). **b**–**h** Naïve T cells were cultured in glucose medium or fructose medium for 3 days under indicated cell culture conditions (*n* = 3). Frequencies of IL-17A^+^CD4^+^ Th17 cells (**b**, **c)** and RORγt^+^CD4^+^ T cells (**d)** in T cells post 3 days culture. The RNA levels of *Il17a* (**e**) and *Rorc* (**f**) in T cells post 3 days culture. **g**, **h** Frequencies of IFN-γ^+^CD4^+^ Th1 cells in T cells post 3 days culture under indicated cell culture conditions. Unpaired two-tailed Student’s *t* tests were used to calculate statistical significance. Data are presented as mean ± SD. *p < 0.05; **p < 0.01; ***p < 0.001; ****p < 0.0001

Since we have determined that, besides glutamine metabolism-dependent mTORC1 activation, high fructose-induced TGF-β activation by driving ROS production is also involved in fructose-induced Th17 cell differentiation (Fig. 7), we next investigated whether metformin could also suppress high fructose-induced Th17 cell generation by suppressing ROS. When naïve T cells were cultured with metformin in media containing fructose and glucose for 24 h, ROS production in the T cells of both groups was significantly suppressed (Fig. 9a). Then naïve T cells were cultured in fructose and glucose media, with or without IL-6, latent TGF-β1, and metformin. The results showed that metformin also reversed Th17 cell differentiation induced by fructose-induced TGF-β activation (Fig. 9b–f). Notably, under these Th17-cell induction conditions, the generation of Th1 cells was still significantly higher in fructose-cultured T cells than in the glucose group (Fig. 9g, h and Supplementary Fig. 10a, b). Consistently, the difference was eliminated after metformin treatment (Fig. 9g, h). Under Th17 cell differentiation conditions, metformin did not significantly affect other T cell subtypes, including Th2 and Tr1 cells (Supplementary Fig. 10c–f). In contrast, Treg cell frequency increased in T cells cultured with fructose medium and treated with metformin (Supplementary Fig. 10g, h). These data showed that metformin can suppress Th17 cell generation by inhibiting fructose-induced TGF-β activation via reducing ROS production.

### Metformin supplementation reverses high fructose consumption-induced T cell inflammation in vivo

**Fig. 10:**
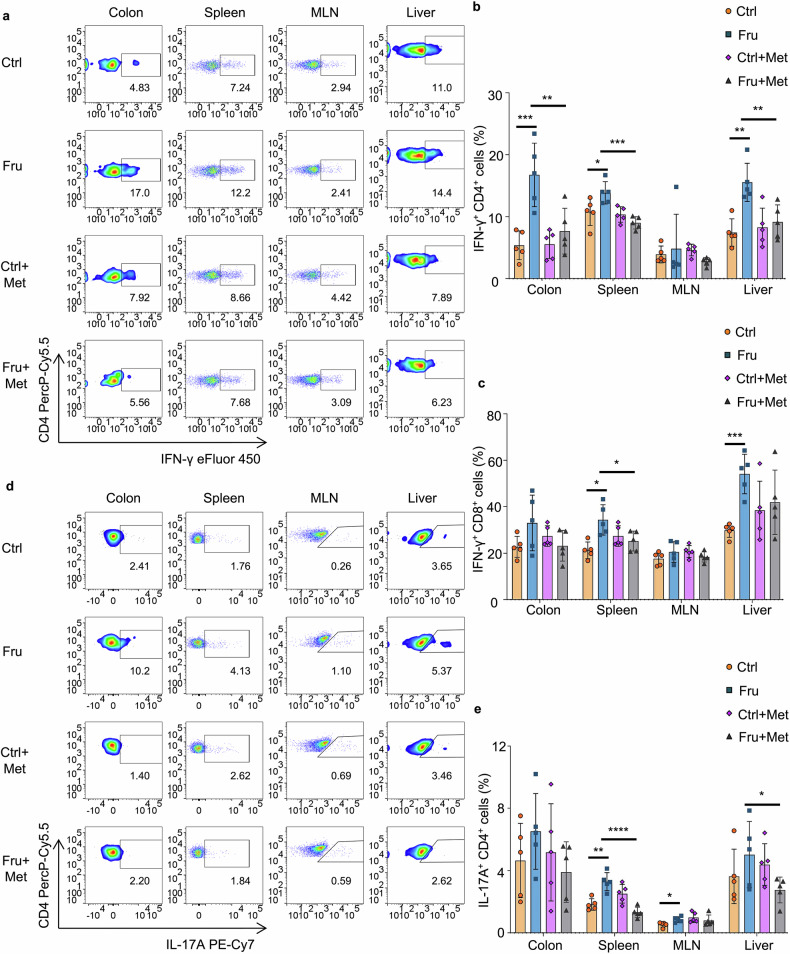
Metformin supplementation inhibits high fructose consumption-induced T cell inflammation in vivo. C57BL/6 mice were treated with control or high fructose water for 8 weeks, and half of these mice were treated with metformin (2.5 mg/mL) during the 5th to 8th weeks (*n* = 5). One of two independent experiments was showed. **a**, **b** Frequencies of IFN-γ^+^CD4^+^ Th1 cells in indicated tissues of the mice. **c** Frequencies of IFN-γ^+^CD8^+^ Tc1 cells in indicated tissues of the mice. **d**, **e** Frequencies of IL-17A^+^CD4^+^ Th17 cells in indicated tissues of the mice. One-way ANOVA (with Tukey’s multiple-comparisons post-tests) was used to calculate statistical significance. Data are presented as mean ± SD. *p < 0.05; **p < 0.01; ***p < 0.001

The findings of in vitro studies encouraged us to explore whether metformin could reverse high fructose consumption-induced T cell inflammation in vivo. First, the mice were fed normal drinking water or water containing 20% fructose for 8 weeks, and then treated these mice with or without metformin through the drinking water since the 5th week (Supplementary Fig. 11a). No significant changes in body weights among the four groups of mice during treatment (Supplementary Fig. 11b). As expected, high fructose consumption increased Th1, Tc1, and Th17 cell immunity in the colon, spleen, MLN, or liver of mice, and metformin supplementation significantly suppressed fructose-induced Th1, Tc1, and Th17 cell generation (Fig. 10a–e and Supplementary Fig. 11c). Metformin supplementation did not affect substantially other T-cell subtypes, including Tr1, Th2, and Treg cells (Supplementary Fig. 11d–f).

**Fig. 11:**
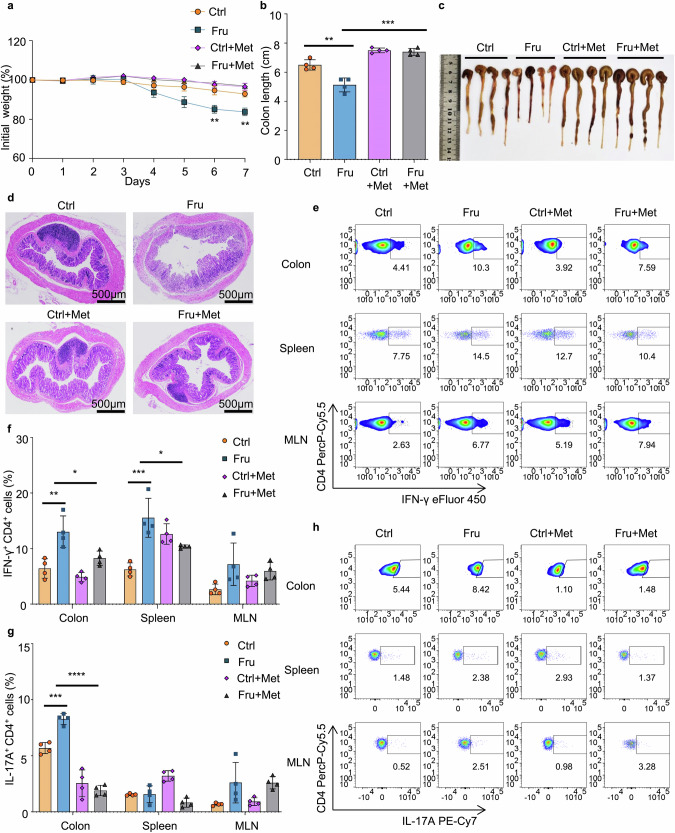
Metformin supplementation suppresses high fructose intake-induced colitis aggravation. C57BL/6 mice were treated with control or high fructose water for 8 weeks, and half of these mice were treated with metformin (2.5 mg/mL) during the 5th to 8th weeks. Then, the DSS-induced colitis model was established to investigate the disease development after fructose water treatment (*n* = 4). One of two independent experiments was shown. **a** Changes in body weight during the colitis induction. **b**, **c** The length and photograph of the colons. **d** H&E-stained colon sections. **e**, **f** Frequencies of IFN-γ^+^CD4^+^ Th1 cells in indicated tissues of the mice. **g**, **h** Frequencies of IL-17A^+^CD4^+^ Th17 cells in indicated tissues of the mice. One-way ANOVA (with Tukey’s multiple-comparisons post-tests) was used to calculate statistical significance. Summary data are presented as mean ± SD. *p < 0.05; **p < 0.01; ***p < 0.001; ****p < 0.0001

To further investigate whether metformin supplementation can reverse high fructose consumption-induced colitis aggravation, the DSS-induced colitis model was established following metformin treatment (Supplementary Fig. 12a). Notably, metformin supplementation indeed reversed high fructose consumption-induced colitis aggravation (Fig. 11a–d). Besides, metformin supplementation also reversed high fructose consumption induced Th1, Tc1, and Th17 cell generation without affecting Treg cells in the DSS-induced colitis model (Fig. 11e–h and Supplementary Fig. 12b–e). These data demonstrated that metformin supplementation can reverse high fructose consumption-induced effector T cell generation in vivo, indicating that metformin could be a promising dietary supplement for suppressing high fructose intake-induced T cell inflammation (Supplementary Fig. 13). Taken together, fructose promotes the differentiation of Th1 and Th17 cells by activating mTORC1 through the glutamine metabolism-dependent pathway, and ROS-induced TGF-β activation is also involved in fructose-induced Th17 cell differentiation (Supplementary Fig. 13).

## Discussion

Several studies have shown a correlation between high fructose intake and non-hereditary diseases, including metabolic diseases and cancer.^2,10,38–40^ We show that high fructose consumption could promote T-cell immunity by inducing Th1 and Th17 cells. We identified that high fructose consumption exacerbates the development of inflammatory bowel disease in two disease models: T cell-transfer colitis and DSS-induced colitis. Although the pathogenesis of these two models of IBD is different, high fructose consumption increases the inflammatory responses of effector T cells in both models. Based on these in vivo findings, we demonstrated that fructose promotes Th1 and Th17 differentiation through glutamine metabolism-dependent mTORC1 activation. We also showed that high fructose-induced TGF-β activation through ROS-dependent pathway is involved in high fructose-induced Th17 cell differentiation. We demonstrated that rapamycin and metformin can effectively inhibit fructose-induced Th1 and Th17 cell differentiation by suppressing mTORC1 activation, while metformin can also restrain fructose-induced Th17 cell generation by inhibiting the activation of latent TGF-β. Finally, we demonstrated that metformin is a promising candidate for reversing T-cell inflammation caused by high fructose consumption. These findings not only reveal the previously unrecognized risk of high fructose intake in disrupting immune homeostasis and exacerbating inflammatory bowel disease by promoting Th1 and Th17 cell generation, but also provide potential therapeutic agents for reversing T-cell immune imbalance caused by long-term high fructose consumption.

A recent study showed that fructose exposure could increase IL-1β production from mononuclear phagocytes in response to LPS stimulation by activating mTORC1.^13^ However, the function of high fructose diet on T-cell immunity remains to be elucidated. Our findings revealed the direct regulation of T-cell inflammation by high fructose consumption through the induction of Th1 and Th17 cell generation. Another study reported that fructose can promote the production of leptin,^15^ which might explain why high fructose water treatment did not increase body weight of the C57BL/6 mice. The liver was considered the primary site of fructose metabolism; however, some recent findings showed that dietary fructose metabolism begins in the small intestinal epithelium.^11,41^ Therefore, we chose IBD models to determine the effects of high fructose consumption on immune responses. By investigating T-cell responses after high fructose water treatment in the two IBD models, we found that high fructose consumption increased Th1 and Th17 cell generation. In contrast, Th2, Tr1, and Treg cells remained unchanged. The DSS-induced colitis is mainly caused by macrophage dysfunction, dysregulation of gut microbiota, and toxic effects on the colon epithelium,^37,42,43^ although T-cell inflammation is also involved. To determine whether the aggravation of colitis induced by high fructose consumption relies mainly on T-cell inflammation, we established the DSS-induced colitis model using nude mice or *Rag1*^−/−^ mice. Our data indicated no difference in colitis development between high fructose water-treated and control nude mice or *Rag1*^−/−^ mice, showing that high fructose consumption did not aggravate colitis in T cell-deficient mice. Taken together, we demonstrated that high fructose consumption can promote Th1 and Th17 cell generation and aggravate IBD. In addition to the in vivo study, we established T cell cultures in a medium containing fructose or glucose. Fructose can directly promote the differentiation of effector T cells, including Th1 and Th17 cells. The discovery that high fructose levels promoted Th1 and Th17 cell generation prompted us to explore their regulatory mechanisms further. Cellular metabolic signaling pathways play crucial roles in regulating the differentiation and function of T cells.^25^ Glycolysis, the main path of hexose metabolism, plays vital roles in cell growth, differentiation, and function.^44^ Surprisingly, we found that fructose could not be used for T-cell glycolysis efficiently and that it suppressed T-cell glycolysis. In the meanwhile, the level of glutamine was significantly increased in fructose-treated T cells. Glutamine, the body’s most abundant free amino acid, is converted to alpha-ketoglutaric acid (αKG) in cells to support the TCA cycle.^45,46^ By treating fructose-cultured T cells with the glutaminase inhibitor, CB839, we determined that fructose-induced Th1 and Th17 cell generation relies entirely on glutaminase metabolism.

Our previous study showed that high glucose can promote Th17 cell differentiation via ROS-mediated activation of latent TGF-β and exacerbate autoimmune diseases.^12^ Meanwhile, long-term fructose intake has also been reported to increase ROS production in rat peripheral blood monocytes.^47^ We found that fructose and glucose induced comparable ROS production in cultured T cells. This encouraged us to investigate whether fructose can induce Th17 cell differentiation through the same mechanism. Our data showed that both high fructose and high glucose induced more Th17 cell generation when T cells were cultured with latent TGF-β1 and IL-6, while either blocking ROS or TGF-β signaling could completely block high sugar-induced Th17 cell generation, indicating that high fructose can also induce Th17 cell generation by promoting the activation of latent TGF-β via ROS-dependent pathway. In addition, fructose induced more Th17 cells than glucose at the same dose, possibly because fructose can promote Th17 cell differentiation by upregulating mTORC1, which requires more in-depth research in the future. On the other hand, Khan et al. showed that high fructose consumption can change the gut microbiome dramatically.^11^ They found that the increase in the abundance of some specific gut microbiomes, including *Achaemophilus mucinalis* and *Bacteroides fragilis*, plays a significant role in high fructose-mediated colitis aggravation and Th17 cell differentiation. Therefore, the impact of high fructose consumption on intestinal immune homeostasis may be complex and requires more in-depth research in the future.

Based on these mechanistic studies, we investigated whether there is an effective drug to reverse or relieve T-cell inflammation induced by high fructose consumption. Since metformin was first shown in 1918 to reduce blood sugar levels, it has gradually become a conventional oral drug for the treatment of T2D.^48,49^ One study showed that metformin can prevent the production of Th17 cell-related pro-inflammatory cytokines.^33^ Moreover, metformin inhibits mTORC1 activation in oral squamous cell carcinoma.^50^ Given the therapeutic effects of metformin in multiple diseases, such as inflammation and diabetes, we attempted to use metformin as a drug to treat T-cell inflammation induced by high fructose intake. We found that metformin suppressed fructose-induced mTORC1 activation and ROS production and reversed Th1 and Th17 cell generation. The findings regarding the effects of metformin in suppressing gut inflammation in a colitis model also support our conclusion.^34^ Besides the mTOR signaling pathway, key enzymes in the glutamine metabolic pathway, such as glutaminase, are also potential targets for targeted therapy of inflammation caused by high fructose intake.

In conclusion, our study revealed that high fructose consumption drives Th1 and Th17 cell generation and aggravates the development of IBD. In terms of metabolism, T cells do not express fructose transporter GLUT5 and cannot effectively utilize fructose. Therefore, high fructose microenvironment reduces glucose level in the internal environment and suppresses T cell glycolysis, leading to a compensatory increase in glutamine metabolism in T cells. Mechanistically, high fructose levels promote Th1 and Th17 cell differentiation by activating mTORC1 via a glutamine metabolism-dependent pathway. Besides, high fructose can also promote Th17 cell generation through ROS-induced activation of latent TGF-β. Metformin can reverse high fructose-induced Th1 and Th17 cell generation via suppressing mTORC1 and reducing ROS-mediated TGF-β activation. Notably, metformin inhibits high fructose consumption-induced T-cell inflammation and colitis aggravation. Our study revealed the adverse effect of high fructose consumption on effector T cell-mediated inflammation, highlighting the importance of controlling fructose intake in the daily diet.

## Materials and methods

### Mice

C57BL/6, BALB/c-nu, and *Rag1*^−/−^ mice were housed in a specific pathogen-free facility at Sichuan University for breeding. Mice aged 6–10 weeks were fed according to a 12-hour diurnal cycle and randomly divided into control and experimental groups. All animal studies were performed in accordance with the guidelines of the Animal Care and Use Committee of West China Hospital, Sichuan University, and were approved by the Animal Care and Use Committee of West China Hospital, Sichuan University (Approval Nos. 2021736A, 20240226111).

### High fructose consumption model of C57BL/6 mice

Six to eight-week-old C57BL/6 mice were randomly assigned to two groups and fed normal drinking water or water containing 20% fructose for 2 months.^51^ After the mice were euthanized, colon, spleen, MLN, and liver tissues were collected for histopathological and immunological analyses. Parts of the colon, spleen, and liver tissues of the mice were fixed, and H&E staining was performed on the sections. The severity of histological damage was evaluated as described previously.^52^ All other tissues were used to prepare single-cell suspensions for flow cytometry.

### DSS-induced colitis model

Six to eight-week-old C57BL/6, BALB/c-nu, or *Rag1*^−/−^ mice were randomly assigned to two groups 2 months before modeling and were fed with or without 20% fructose water. During colitis induction, mice were fed 3% DSS for 7 days, and body weight changes were measured daily.^53^ After the mice were euthanized, the colon, spleen, and MLN were subjected to histopathological and immunological analyses. The Inflammatory scoring criteria for colitis were established as previously described.^12^

### T cell-transfer colitis model

The model was established as previously described.^12,54^ Two days before T cell transfer, *Rag1*^−/−^ mice were fed with or without 20% fructose water. Subsequently, the mice were injected with CD4^+^CD25^-^CD45RB^hi^ T cells sorted from the spleen of C57BL/6 mice (0.4 × 10^6^ cells/mouse). During the modeling process, weight changes in the mice were measured every 2 to 4 days to monitor the development of colitis. After the mice were euthanized, the colon, spleen, and MLN were subjected to histopathological and immunological analyses.

### Metformin treatment model

C57BL/6 mice aged 8–10 weeks were randomly assigned to four groups and treated with or without 20% fructose water for 8 weeks. During the 5th to 8th week, two groups (one regular drinking water- and one fructose water-treated group) were supplemented with metformin treatment (2.5 mg/mL).^50^ During the modeling process, weight changes of the mice were measured every week. After the mice were euthanized, the colon, spleen, MLN, and liver were collected for histopathological and immunological analyses. For colitis model investigation, the DSS-induced colitis model was established after fructose water treatment.

### Isolation of colonic lamina propria lymphocytes (LPLs)

Colonic lamina propria cells were separated as previously described.^12,55^ Intraepithelial lymphocytes (IELs) were separated using a magnetic stirrer in DMEM containing 5% FBS, DTT (5 mM, ThermoFisher), and EDTA (1 mM, Solarbio). Then the remaining tissues were treated with Librase TL (0.2 mg/mL, Roche) and DNase I (0.5 mg/mL, Aladdin) for digestion at 37 °C with vigorous shaking in the absence of FBS. The tissues were mashed to cell suspension and filtered through a 70 μm cell filter before use.

### Serum fructose content determination

The mice were anesthetized, and blood was collected through the ocular vein. Serum was collected by centrifugation at a relative centrifugal force of 14000 × *g* for 10–15 min. Operate in accordance with the instructions of the fructose content measurement kit and analyze the fructose content in the serum.

### Cell cultures

Complete DMEM (cDMEM) was prepared for all cell cultures by adding 25 mM glucose or fructose to glucose-free DMEM (ThermoFisher, 11966025). CD4^+^CD25^-^CD62L^+^ naïve T cells sorted from C57BL/6 mice were cultured in 48-well plates (4 × 10^5^ cells/mL) in the presence of plate-bound anti-mouse CD3 (1.5 μg/mL, Bio X Cell) and soluble anti-mouse CD28 (1.5 μg/mL, Bio X Cell). For Th1 cell induction, T cells were induced with recombinant mouse IL-12 (10 ng/mL, PEPROTECH). For Th17 cell induction, T cells were induced with recombinant human TGF-β1 (2 ng/mL, PEPROTECH) plus recombinant mouse IL-6 (50 ng/mL, PEPROTECH). For TGF-β activation experiments, T cells were cultured with recombinant mouse IL-6, with or without recombinant human latent TGF-β1 (10 ng/mL, R&D Systems). TGF-β signaling was blocked using anti-TGF-β (50 μg/mL, Bio X Cell) and SB431542 (5 μM, Selleck.cn). NAC (10 mM, MedChemExpress) was used to scavenge ROS. Glutamine metabolism pathway was blocked using a glutaminase inhibitor CB839 (1 μM, Selleck Chemicals). *Gls* gene encodes for glutaminase, which was deleted in cultured T cells using the CRISPR/CAS9 system. The mTOR pathway was blocked using rapamycin (100 nM; Selleck Chemicals) and metformin (5 mM, Selleck Chemicals). Cells were cultured at 37 °C, 5% CO_2_, and collected for flow cytometry analysis 24 h or 3 days later.

### Mutation of Gls gene using CRISPR/CAS9 system

CD4^+^CD25^-^CD62L^+^ naïve T cells sorted from C57BL/6 mice were cultured in the presence of plate-bound anti-mouse CD3 (1.5 μg/mL, Bio X Cell) and soluble anti-mouse CD28 (1.5 μg/mL, Bio X Cell) for 24 h. The *Gls* sgRNA (1 µg/µL) and Cas9 mRNA (1 µg/µL) were simultaneously introduced into T cells using the ProteanFect™ CRISPRMax Transfection Kit (PT06). Then the T cells were re-cultured using the original culture supernatant in the presence of indicated induction cytokines for another 3 days. The *Gls*-targeting sgRNA (target sequence: CCGCAGGACGGCTCCGACGG) was purchased from GenScript.

### RNA-Seq data analyses

RNA-Seq experiments were performed using total RNAs from naïve T cell cultures. Trimmomatic (version 0.36) was used to filter the raw sequencing data. The STRA software (version 2.5.3a) with default parameters was used to map the clean data to the reference genome mm10. Feature counts (Subread-1.5.1) were used to count the reads mapped to the exon regions of each gene. The R package DESeq2 was used for differential expression analysis of genes. Genes with fold-change greater than two and an adjusted *p* value < 0.05 were determined as genes that were significantly differentially expressed.

### Non-target metabolomics analysis

Naïve T cells were cultured in cDMEM containing glucose or fructose for 3 days as previously described. Cultured cells were harvested and frozen in a −80 °C refrigerator. Zhongke New Life Biotechnology Co., Ltd (Shanghai, China) performed the non-targeted metabolomic analysis. First, the samples were slowly thawed on ice. Then, an appropriate amount of sample was added to the pre-cooled methanol/acetonitrile/aqueous solution (2:2:1, v/v). Third, vortex mixed, ultrasonicated at low temperature for 30 min. Fourth, left standing at −20 °C for 10 min. Fifth, centrifuged at 14,000 × g and 4 °C for 20 min, and then vacuum dried. For mass spectrometry analysis, the samples were dissolved again in 100 μL of acetonitrile solution (acetonitrile: water = 1:1, v/v), swirled, centrifuged at 14,000 × g and 4 °C for 15 min, and the supernatant was collected for analysis.

### Seahorse real-time cell metabolic analysis

The ECAR and OCR of fructose-treated CD4^+^ T cells were analyzed with a Seahorse XFe96 extracellular flux analyzer. First, naïve T cells (4 × 10^5^ cell/mL) were cultured in cDMEM containing 25 mM fructose or glucose for 24 h. The cultured cells were then inoculated in Cell-Tak-coated microplates to allow immediate adhesion, spun down, and incubated at 37 °C for half an hour to remove CO_2_ in culture media. After that, the Seahorse XF Cell Mito Stress Test was run for metabolic analysis. To observe the glycolytic status of the different sugars, T cells were incubated in the FBS-free medium containing 11 mM glucose or fructose (using the same sugar for the indicated groups) during the Seahorse XF Cell Mito Stress Test.

### Quantification of western blots

Cells were lysed in a lysis buffer for western blotting. The samples were kept on ice for 30 min and centrifuged at 12,000 × *g* for 20 min to collect the supernatants. Sample buffer was added and boiled for five to ten minutes to denature the samples. The samples were separated and transferred to the PVDF membrane by SDS-PAGE. The membrane was treated with 5% bovine serum albumin (Sigma) at 25 °C for one hour and then incubated overnight with specific antibodies at 4 °C. The membrane was incubated with a secondary antibody at 25 °C for one hour after three times wash with TBST. Enhanced chemiluminescence (ECL, Beyotime) was used to quantify the protein expression.

### Real-time quantitative PCR (Q-PCR)

Total RNA was isolated from the samples using a Total RNA Rapid Extraction Kit (JIANSHI BIOTECH) and was reverse-transcribed into cDNA for long-term preservation. The highly specific Q-PCR reagent TB Green® Premix Ex Taq™ II (Tli RNaseH Plus) (Takara) was used for running Q-PCR. Results were generated using a comparative threshold cycle (ΔCt) and normalized to the *Hprt* gene. The sequences of Q-PCR primers can be found in the Supplementary Table 1.

### Flow cytometry

Dead cells were labeled using the Zombie Yellow Fixable Viability Kit (BioLegend) by incubating at room temperature for 10 min in the dark. Appropriate fluorescent antibodies were coupled by incubating with cells at 4 °C for 20 min to stain cell surface markers. Cells were fixed with the Foxp3/transcription factor staining buffer set (ThermoFisher), permeabilized for one hour or more according to the manufacturer’s instructions, and then stained with antibodies against transcription factors. Cells were cultured in cDMEM with Golgi-Plug (1:1000 dilution, BD Biosciences), PMA (5 ng/mL, Sigma), and ionomycin (1 μg/mL, Aladdin) at 37 °C for 4 h before the intracellular cytokine staining, fixed and permeabilized for 20 min using the BD Cytofix/Cytoperm fixation/ Permeabilization Solution Kit (BD Biosciences), and then stained with the intracellular cytokine antibodies. T cells were collected and incubated with PBS solution containing 5 μM H2DCFDA (Medchemexpress) at 37 °C for 30 min in the dark to determine total ROS production. Samples were collected on a Beckman CytoFLEX S or a BD LSRFortessa. Data were analyzed using FlowJo 10.6.2 software. For details of antibodies and reagents, please see the Supplementary Table 2.

### Statistical analysis

Data were statistically analyzed using GraphPad Prism version 9. Unless otherwise specified, unpaired two-tailed Student’s *t* test was used for the comparison between two groups, and one-way ANOVA (with Tukey’s multiple-comparison post-tests) was used for the comparisons between more than two groups. The values are expressed as mean ± SD. The values *p* < 0.05 were considered statistically significant.

## Supplementary information

Supplementary_Materials_1

Supplementary_Materials_2

Supplementary_Materials_3

## Data Availability

The raw sequence data and non-target metabolomic analysis data of the project have been deposited in the Genome Sequence Archive (GSA) in the National Genomics Data Center and are accessible through accession code CRA018862 (https://ngdc.cncb.ac.cn/gsa/search?searchTerm=CRA018862) and OMIX007324 (https://ngdc.cncb.ac.cn/omix/releaseList). All data supporting the findings of this research are available within this manuscript and its supplementary information files.
