# Supplementary material for: High fructose consumption aggravates inflammation by promoting effector T cell generation via inducing metabolic reprogramming
Source: Signal Transduct Target Ther. 2025 Aug 26;10:271. doi: 10.1038/s41392-025-02359-9 (PMC12379281; doi:10.1038/s41392-025-02359-9)
Supplement: Supplementary file 1 — Supplementary_Materials_1 [file 41392_2025_2359_MOESM1_ESM.docx]

Supplementary Materials for

High fructose consumption aggravates inflammation by promoting effector T cell generation via inducing metabolic reprogramming

Xiao Ma^#1,2^, Jiao Chen^#3^, Fang Wang^#4^, Xinzou Fan^1^, Zhenhong Li^1^, Hantian Liang^1^, Hao Cheng^1^, Fang Nan^1^, Yubin Lin^1^, Xiaoshuang Song^1^, Jianan Zhang^1^, Fan Gao^1^, Wei Zhang^1,2^, Wenwen Jin^5^, Huiyuan Zhang^2^, Jiyu Tong^6^, Hong Jiang^7^, Xikun Zhou^1^, Qiang Zou^8^, Hongbo Hu^2^, Aiping Tong^1,*^, WanJun Chen^5,*^, Dunfang Zhang^1,2,*^

Correspondence to: [izdf@163.com](mailto:izdf@163.com) (D.Z.); [wchen@nih.gov](mailto:wchen@nih.gov) (W.J.C); [aipingtong@scu.edu.cn](mailto:aipingtong@scu.edu.cn) (A.T.).

**This PDF file includes:**

Figures. S1 to S13

Tables S1 to S2

**Other Supplementary Materials for this manuscript include the following:**

Original western blots (separate file)

Gating strategies for the flow cytometry data (separate file)

Figure. S1.


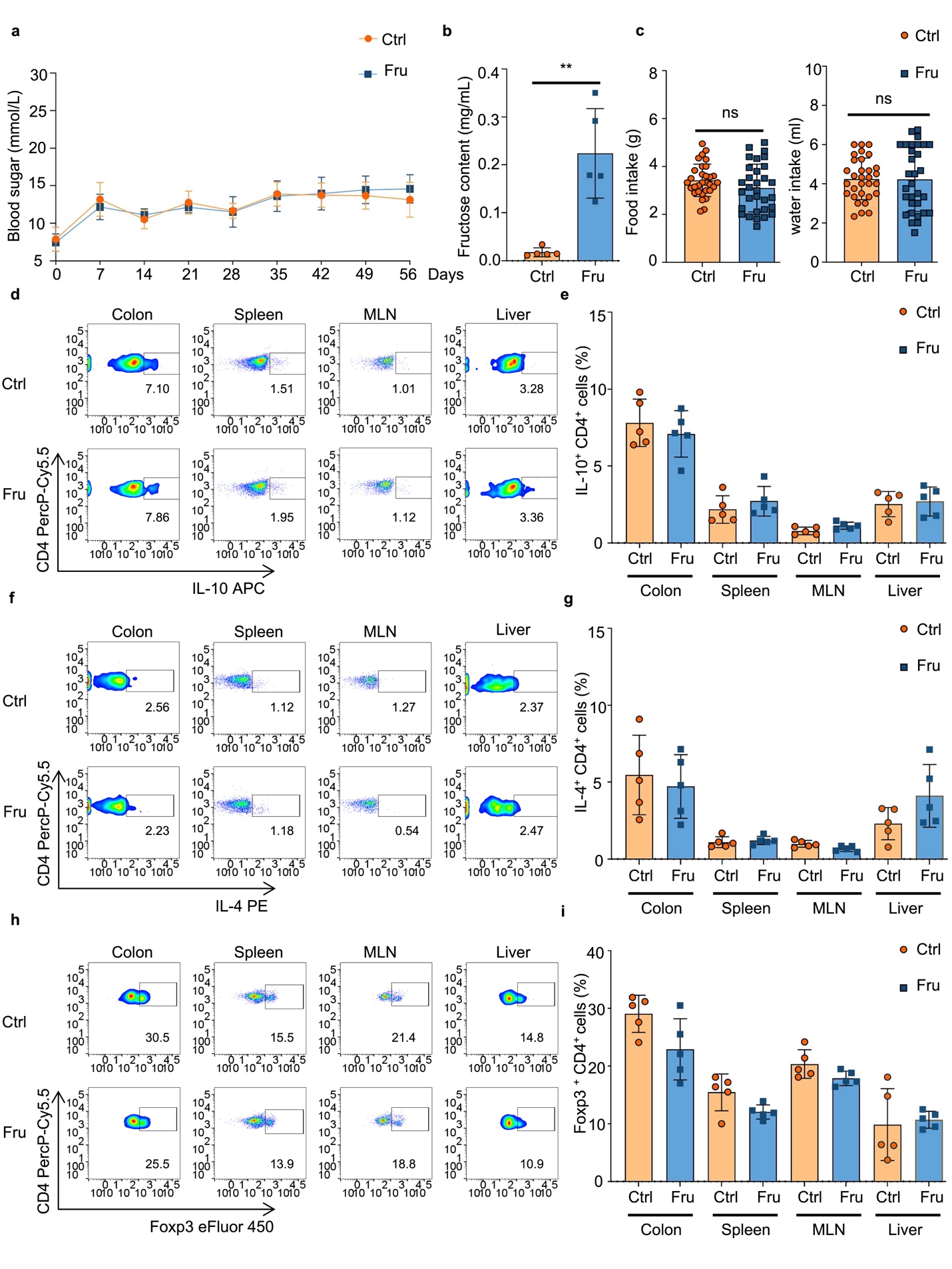


**Supplementary Fig. 1.** High fructose consumption promotes Th1 and Th17 cell-mediated immunity. C57BL/6 mice were treated with regular drinking water or 20% fructose water for two months, and the status of T cell immune responses was investigated. (**a**) Changes in blood sugar levels of the indicated mice (n = 10 mice per group). (**b**) Serum fructose levels in the indicated mice (n = 5 mice per group). (**c**) The mice's daily diet and water intake were measured every three days. (**d**–**i**) Frequencies of IL-10^+^CD4^+^T (Tr1) (**d**, **e**), IL-4^+^CD4^+^ T (Th2) (**f**, **g**), and Foxp3^+^CD4^+^ regulatory T (Treg) (**h**, **i**) cells in the colon, spleen, mesenteric lymph nodes (MLN), and liver of mice. Data are representative of two independent experiments (**b**–**h**) or pooled from two independent experiments (**a**). Unpaired two-tailed Student’s t-tests were used to calculate statistical significance. Summary data are presented as mean ± SD. **p < 0.01.

Figure. S2.


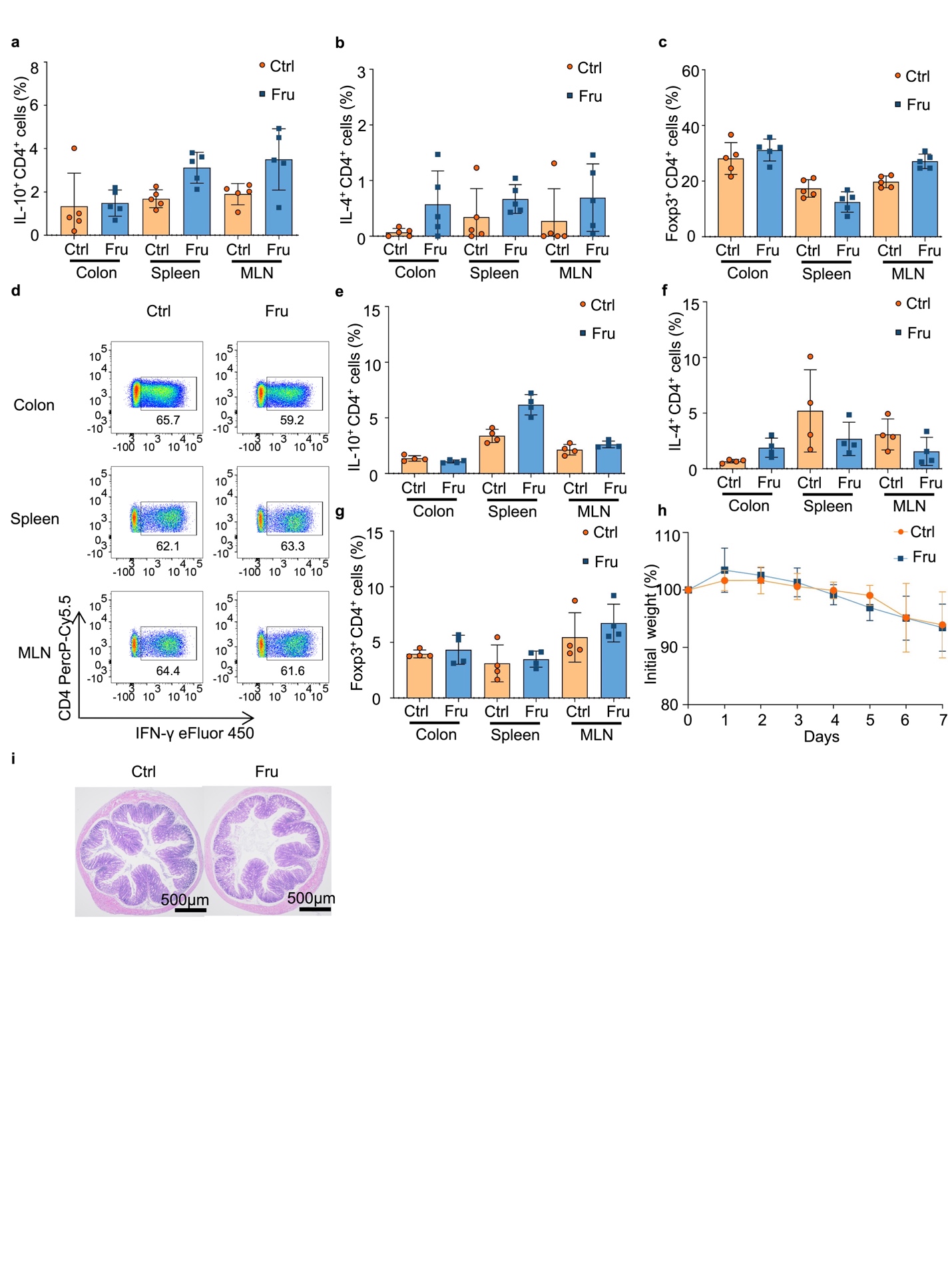


**Supplementary Fig. 2.** High fructose consumption exacerbates inflammatory bowel disease by promoting Th1 and Th17 cell-mediated immunity in two disease models. (**a**–**c**) C57BL/6 mice were treated with regular drinking or 20% fructose water for two months, and a DSS-induced colitis model was established to investigate the disease development (n = 5 mice per group). Bar graphs plots showing the frequencies of Tr1 cells (**a**), Th2 cells (**b**), and Treg cells (**c**) in the colon, spleen, and MLN of DSS-induced colitis mice. (**d**–**g**) *Rag1^-/-^* mice transferred with CD4^+^CD25^-^CD45RB^hi^ T cells were treated with regular drinking or 20% fructose water and then examined for colitis development (n = 4 mice per group). Bar graphs and representative flow cytometry plots showing the frequencies of Th1 (**d**), Tr1 (**e**), Th2 (**f**), and Treg (**g**) cells in the colon, spleen, and MLN of T cell transfer colitis mice. (**h** and **i**) Nude mice were treated with regular drinking or 20% fructose water for two months, and a DSS-induced colitis model was established to identify the disease development. (**h**) The changes in body weight during the colitis induction (n = 4 mice per group). (**i**) Representative histology images of colon sections. Scale bars, 500 μm. Data are representative of two independent experiments. Summary data are presented as mean ± SD.

Figure. S3.


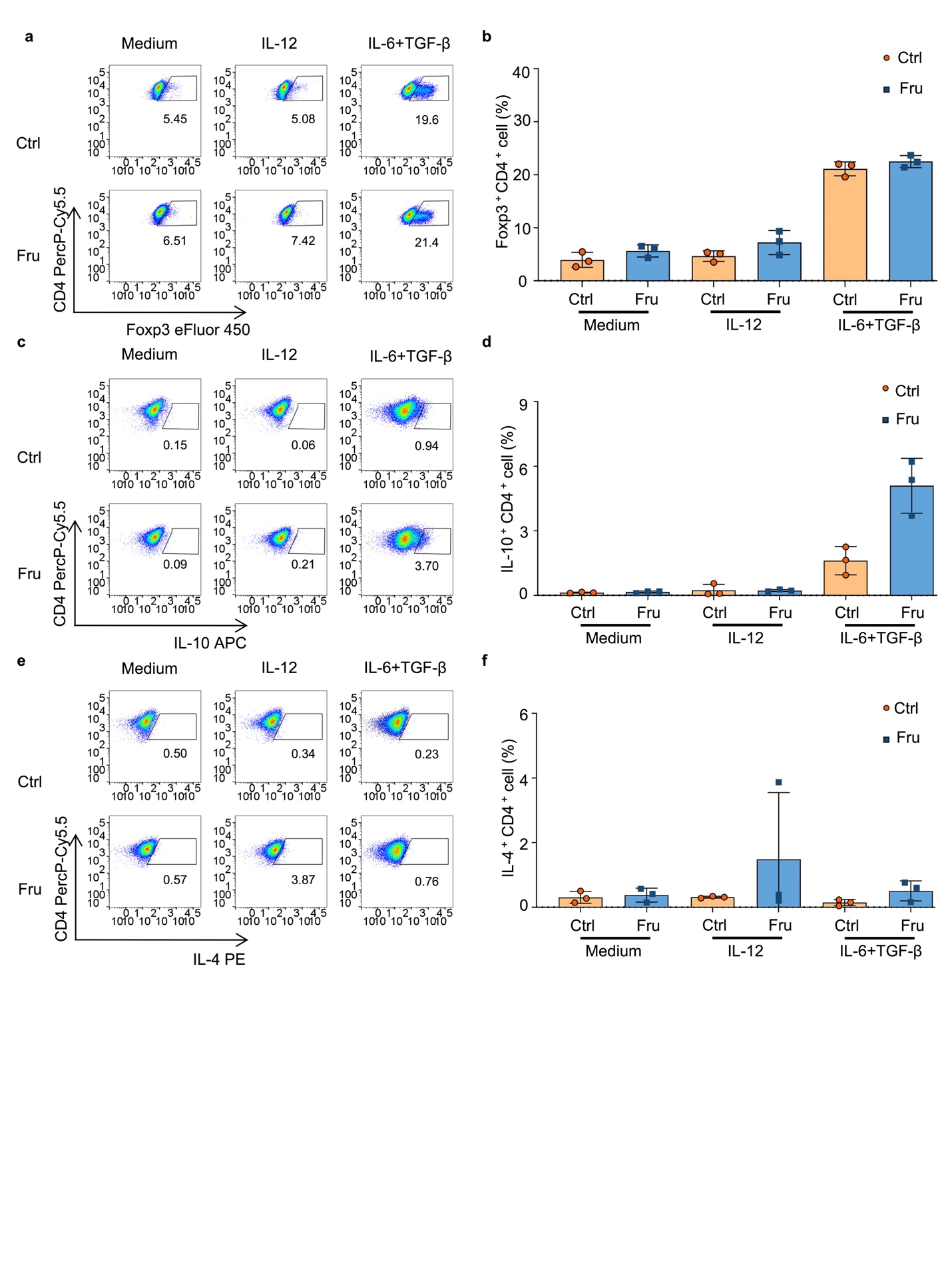


**Supplementary Fig. 3**. Fructose directly promotes differentiation of Th1 and Th17 cells *in vitro.* CD4^+^CD25^-^CD62L^+^ naïve T cells were cultured in complete DMEM containing 25 mM glucose or 25 mM fructose, with plate-bound anti-mouse-CD3 (1.5 μg/mL), soluble anti-mouse-CD28 (1.5 μg/mL), with or without indicated cytokines. Th1 cells were induced with recombinant mouse IL-12 (10 ng/mL), and Th17 cells were induced with recombinant human TGF-β1 (2 ng/mL) and recombinant mouse IL-6 (50 ng/mL). Cells were cultured at 37 ℃, 5% CO_2_ for three days. (**a**–**f**) Frequencies of Treg (**a**, **b**), Tr1 (**c**, **d**), and Th2 (**e**, **f**) cells in indicated groups. Data are representative of three independent experiments (**a**, **c**, and **e**) or pooled from three independent experiments (**b**, **d**, and **f**). Summary data are presented as mean ± SD.

Figure. S4.


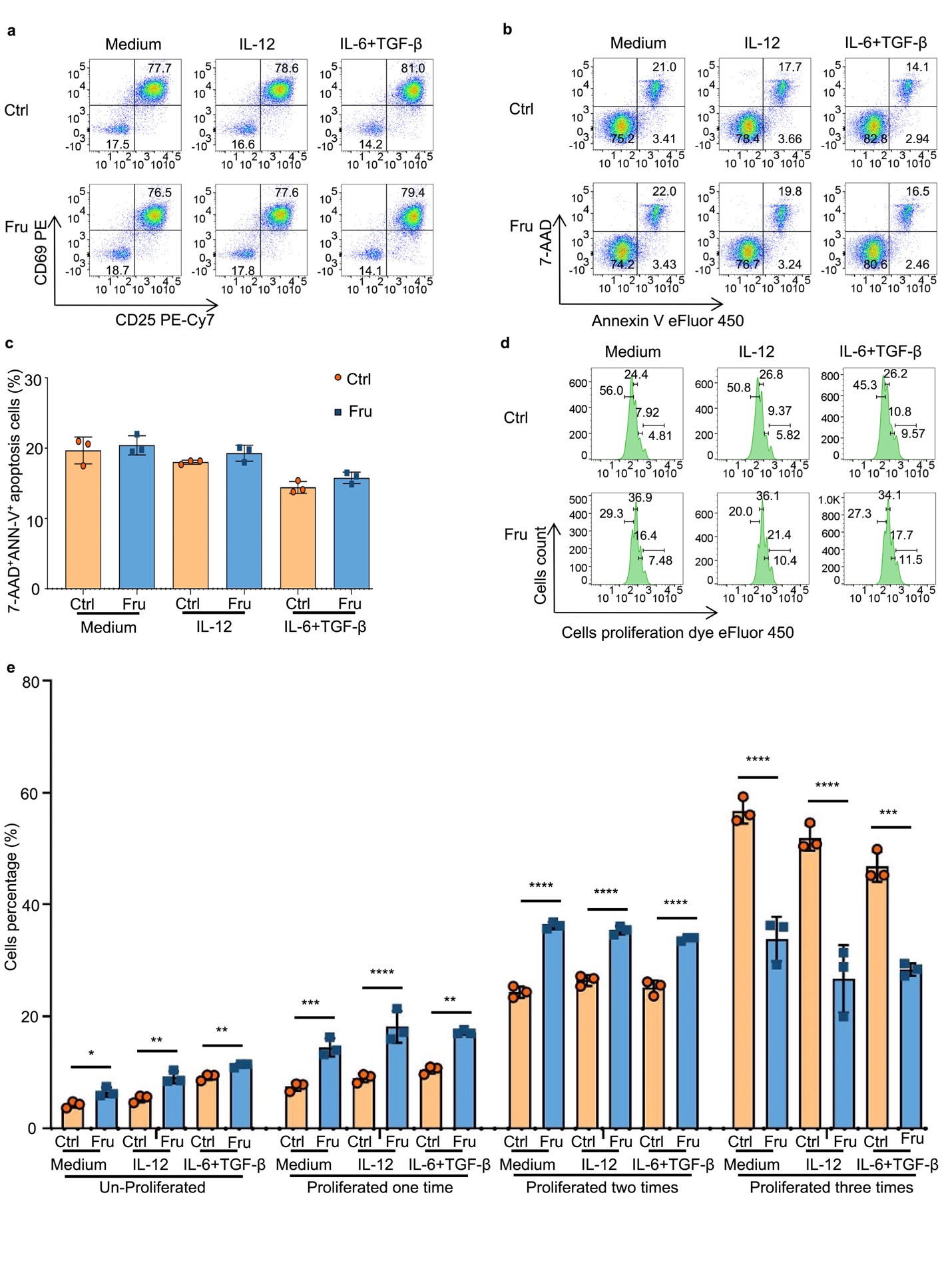


**Supplementary Fig. 4.** Th1 and Th17 cell differentiation promoted by fructose is not through the regulation of T cell activation. CD4^+^CD25^-^CD62L^+^ naïve T cells were cultured in cDMEM containing 25mM glucose or 25 mM fructose, with plate-bound anti-mouse-CD3 (1.5 μg/mL), soluble anti-mouse-CD28 (1.5 μg/mL), with or without indicated cytokines. Th1 cells were induced with IL-12 (10 ng/mL), and Th17 cells were induced with TGF-β1 (2 ng/mL) and IL-6 (50 ng/mL). Cells were cultured at 37 ℃, 5% CO_2_ for 24 hours or three days. (**a**) Frequencies of CD69^+^CD25^+^ activated T cells in T cells cultured under induction conditions of Th0, Th1, and Th17 cells for 24 hours. (**b** and **c**) Apoptosis of T cells cultured under induction conditions of Th0, Th1, and Th17 cells for 24 hours. (**d** and **e**) The proliferation of T cells cultured under induction conditions of Th0, Th1, and Th17 cells for three days. Data are representative of three independent experiments (**b** and **d**) or are pooled from three independent experiments (**c** and **e**). Summary data are presented as mean ± SD. *p < 0.05, **p < 0.01, ***p < 0.001 and ****p < 0.0001, unpaired two-tailed Student’s t-tests.

Figure. S5.


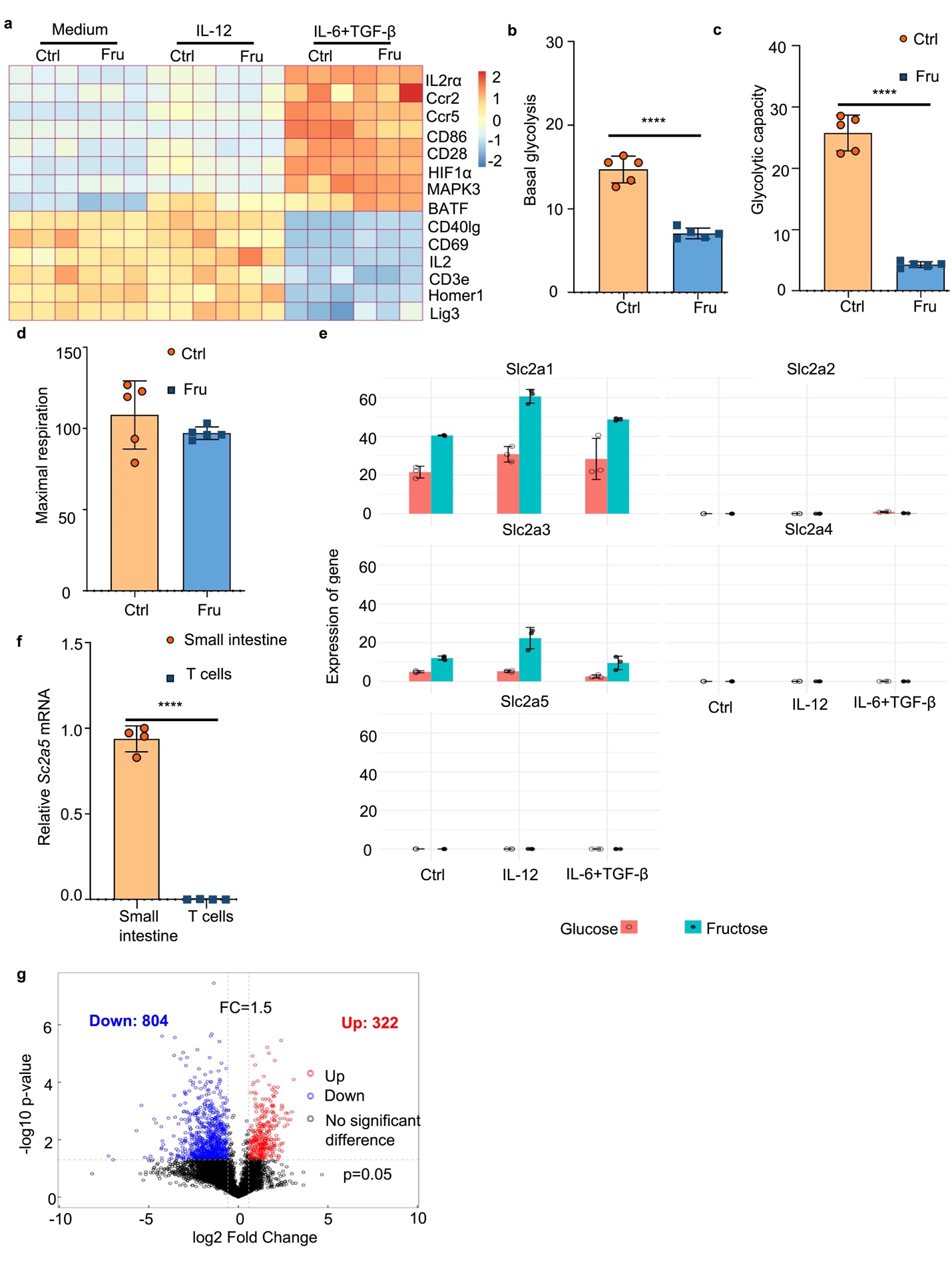


**Supplementary Fig. 5.** T cells cannot transport and utilize fructose effectively. (**a**) Expression of genes related to T-cell activation and differentiation between the fructose- and glucose-treated T cells in Th0, Th1, and Th17 induction conditions analyzed by RNA-seq (n = 3 per group). (**b**–**d**) Basal glycolysis, glycolytic capacity, and maximal respiration of CD4^+^ T cells cultured in glucose or fructose medium for 24 hours were determined by Seahorse XF Cell Mito Stress Test Kit. (**e**) Expression of *Slc2a1*, *Slc2a2*, *Slc2a3*, *Slc2a4*, and *Slc2a5* in fructose- and glucose-treated T cells analyzed by RNA-seq (n = 3 per group). (**f**) The RNA level of *Slc2a5* in mouse small intestine and sorted CD4^+^ T cells. (**g**) A volcanic map of differences between all metabolites detected in the fructose- and glucose-treated T cells (In the figure, the horizontal coordinate is the logarithm of log_2_ of the Fold Change, and the vertical coordinate is the logarithm of -log_10_ of the significance p-value). Significant difference metabolites: Metabolites meeting FC > 1.5 and p-value < 0.05 are shown in red, metabolites meeting FC < 0.67 and p-value < 0.05 are shown in blue, and metabolites with no significant differences are shown in gray) (n = 4 per group). Data are representative of three independent experiments (**b**–**d**) or are pooled from at least three independent experiments (**a**, **e**–**g**). Unpaired two-tailed Student’s t-tests were used to calculate statistical significance. Summary data are presented as mean ± SD. ****p < 0.0001.

Figure. S6.


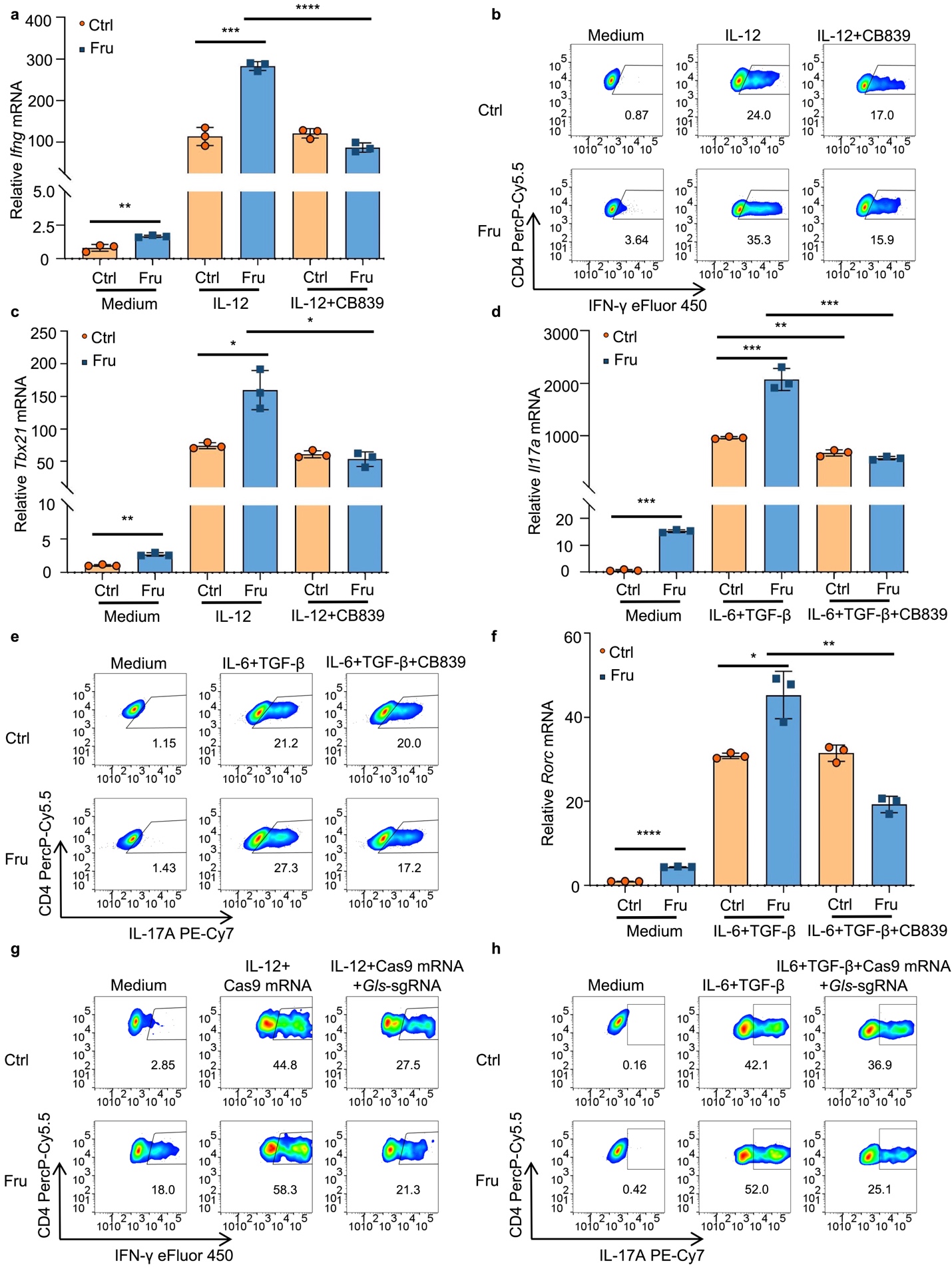


**Supplementary Fig. 6.** Fructose promotes Th1 and Th17 cell differentiations through a glutamine metabolism-dependent pathway. CD4^+^CD25^-^CD62L^+^ naïve T cells were cultured in cDMEM containing 25 mM glucose or fructose, with plate-bound anti-mouse-CD3 (1.5 μg/mL), soluble anti-mouse-CD28 (1.5 μg/mL), with or without indicated cytokines and CB839. Th1 cells were induced with mouse IL-12 (10 ng/mL), and Th17 cells were induced with human TGF-β1 (2 ng/mL) plus mouse IL-6 (50 ng/mL), and glutamine metabolism pathway was blocked with a glutaminase inhibitor CB839 (1 μM) or by *Gls* gene knockout using CRISPR/CAS9 system. Cells were cultured at 37 ℃, 5% CO_2_ for three days. (**a**) The RNA level of *Ifng* under indicated cell culture conditions. (**b**) Frequencies of IFN-γ^+^ CD4^+^ Th1 cells under indicated cell culture conditions. (**c**) The RNA level of *Tbx21* under indicated cell culture conditions. (**d**) The RNA level of *Il17a* under indicated cell culture conditions. (**e**) Frequencies of IL-17A^+^ CD4^+^ Th17 cells under indicated cell culture conditions. (**f**) The RNA level of *Rorc* under indicated cell culture conditions. (**g** and **h**) Frequencies of IFN-γ^+^ CD4^+^ Th1 cells (**g**), IL-17A^+^ CD4^+^ Th17 cells (**h**) in control T cells or *Gls* mutant T cells under indicated cell culture conditions. Data are representative of three independent experiments (**b**, **e**, **g**, **h**) or are pooled from three independent experiments (**a**, **c**, **d**, **f**). Unpaired two-tailed Student’s t-tests were used to calculate statistical significance. Summary data are presented as mean ± SD. *p < 0.05; **p < 0.01; ***p < 0.001; ****p < 0.0001.

Figure. S7.


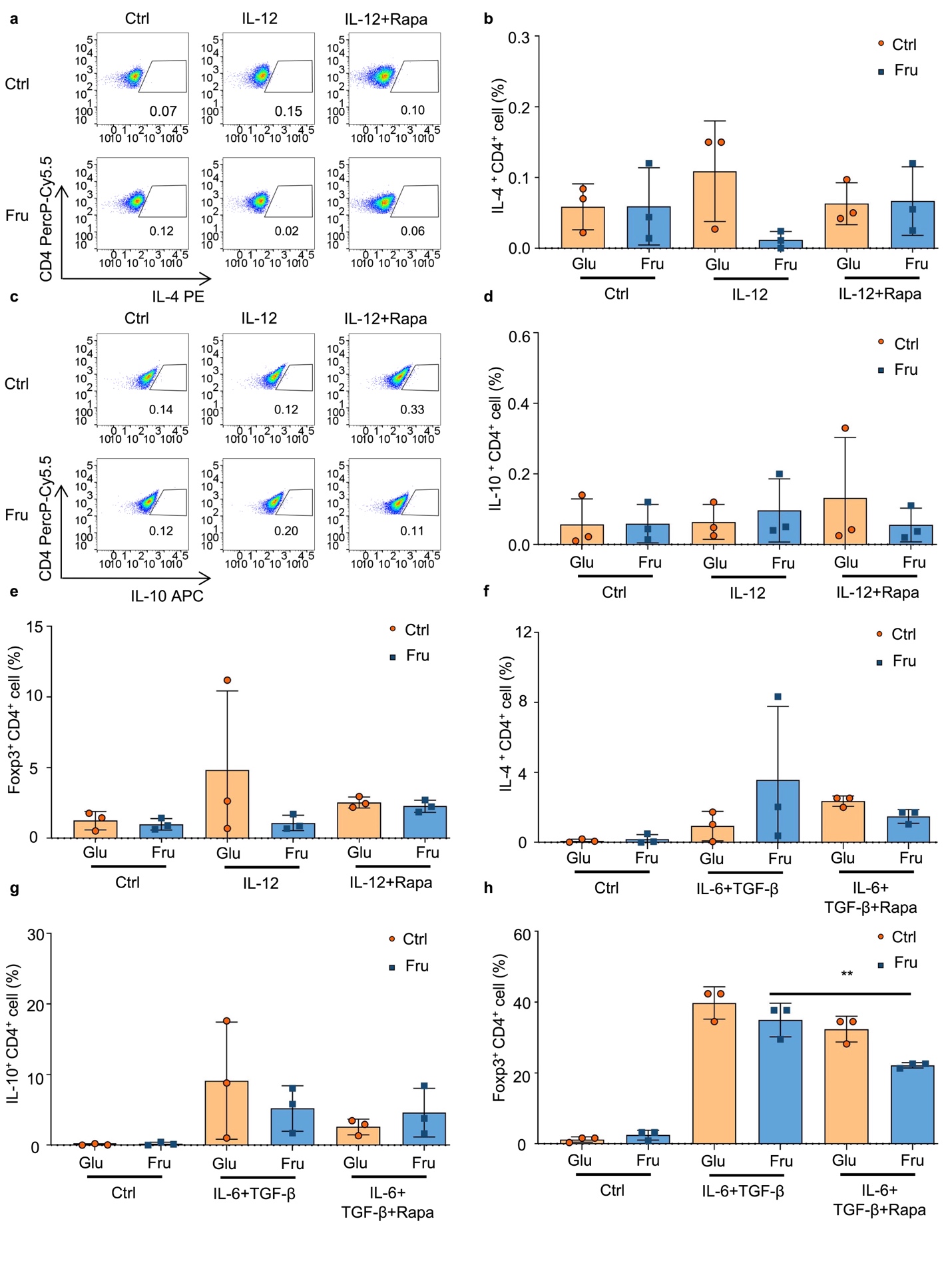


**Supplementary Fig. 7.** Fructose promotes Th1 and Th17 cell differentiation via glutamine metabolism-dependent mTORC1 activation. CD4^+^CD25^-^CD62L^+^ naïve T cells were cultured in cDMEM containing 25 mM glucose or fructose, with plate-bound anti-mouse-CD3 (1.5 μg/mL) and soluble anti-mouse-CD28 (1.5 μg/mL), with or without indicated cytokines and reagent. Th1 cells were induced with mouse IL-12 (10 ng/mL), and Th17 cell were induced with human TGF-β1 (2 ng/mL) plus mouse IL-6 (50 ng/mL), and mTOR pathway was blocked with rapamycin (100 nM). Cells were cultured at 37 ℃, 5% CO_2_ for three days. (**a**–**l**) Frequencies of Th2 cells (**a**, **b**, and **f**), Tr1 cells (**c**, **d**, and **g**) and Foxp3^+^Treg cells (**e** and **h**) in T cells cultured for three days under indicated cell culture conditions. Data are representative of three independent experiments (**a** and **c**) or are pooled from three independent experiments (**b**, **d**, **e**–**h**). Unpaired two-tailed Student’s t-tests were used to calculate statistical significance. Summary data are presented as mean ± SD. **p < 0.01.

Figure. S8.


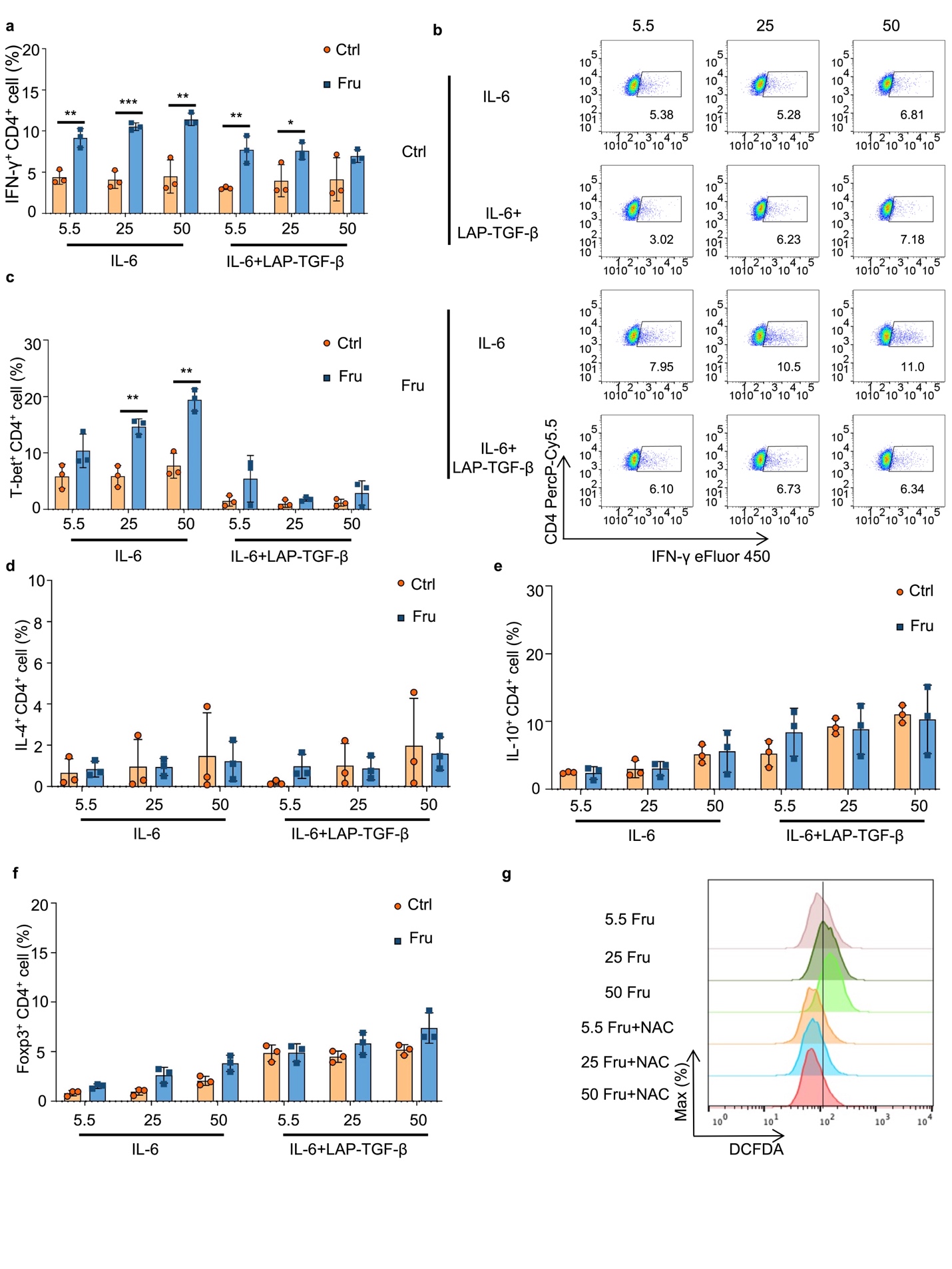


**Supplementary Fig. 8.** High fructose-induced TGF-β activation is involved in fructose-induced Th17 cell differentiation. CD4^+^CD25^-^CD62L^+^ naïve T cells were cultured for three days in cDMEM containing 5.5, 25, and 50 mM glucose or fructose, with plate-bound anti-mouse-CD3 (1.5 μg/mL) and soluble anti-mouse-CD28 (1.5 μg/mL). Th17 cells were induced with mouse IL-6 (50 ng/mL), with or without human latent TGF-β1 (10 ng/mL). (**a**, **b**) Frequencies of IFN-γ^+^CD4^+^ Th1 cells in T cells cultured under indicated cell culture conditions. (**c**) Frequencies of T-bet^+^CD4^+^ T cells in T cells cultured under indicated cell culture conditions. (**d**) Frequencies of IL-4^+^CD4^+^ Th2 cells in T cells cultured under indicated cell culture conditions. (**e**) Frequencies of IL-10^+^CD4^+^ Tr1 cells in T cells cultured under indicated cell culture conditions. (**f**) Frequencies of Foxp3^+^ Treg cells in T cells cultured under indicated cell culture conditions. (**g**) CD4^+^CD25^-^CD62L^+^ naïve T cells were cultured in cDMEM containing 5.5, 25, and 50 mM glucose or fructose for 24 hours, with plate-bound anti-mouse-CD3 (1.5 μg/mL) and soluble anti-mouse-CD28 (1.5 μg/mL), with or without NAC (10 mM), and then the production of ROS was determined. Data are representative of three independent experiments (**b** and **g**) or are pooled from three independent experiments (**a**, **c**–**f**). Unpaired two-tailed Student’s t-tests were used to calculate statistical significance. Summary data are presented as mean ± SD. *p < 0.05; **p < 0.01; and ***p < 0.001.

Figure. S9.


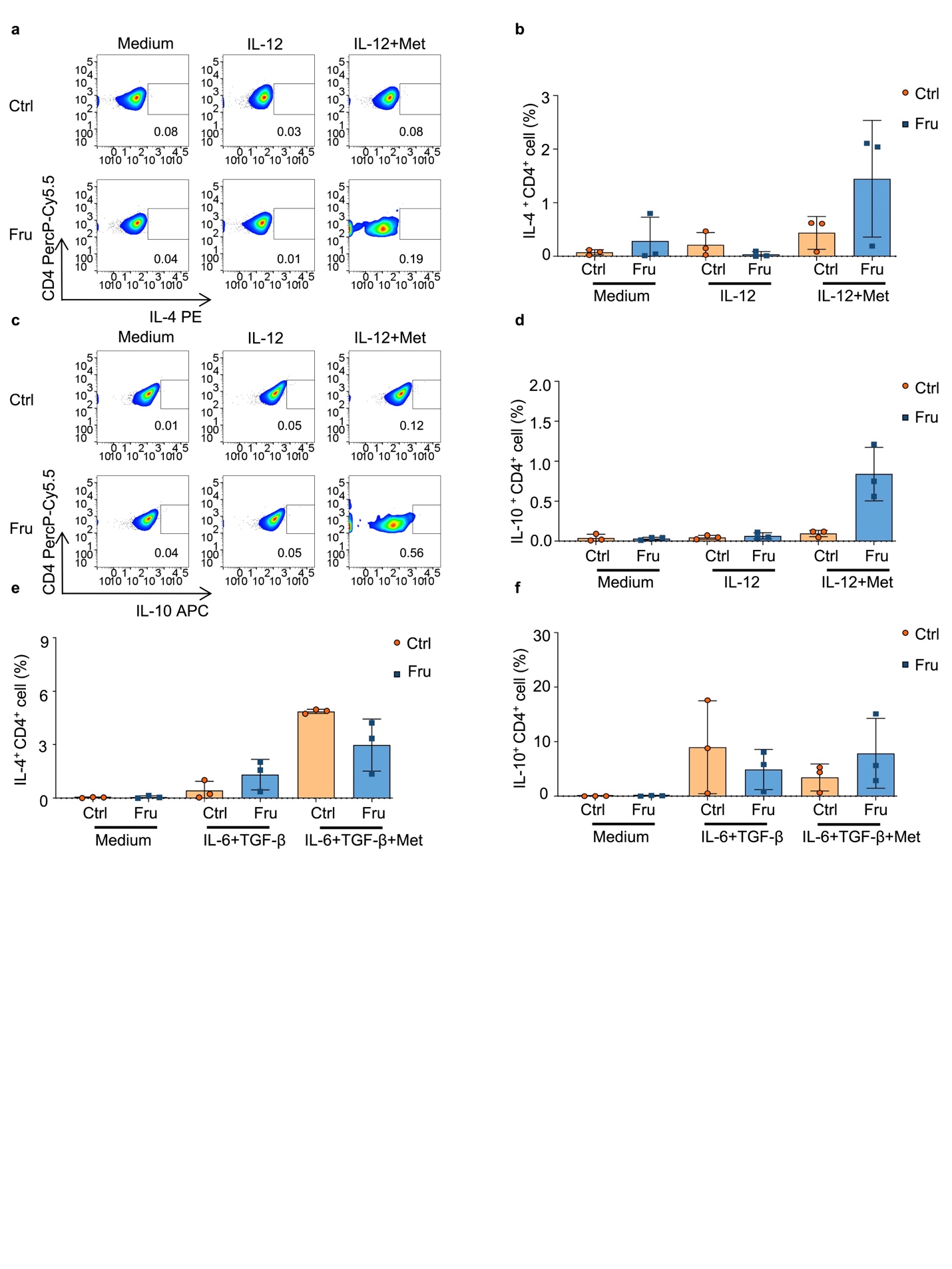


**Supplementary Fig. 9.** Metformin inhibits fructose induced Th1 and Th17 cell differentiations by suppressing mTORC1. CD4^+^CD25^-^CD62L^+^ naïve T cells were cultured in cDMEM containing 25 mM glucose or fructose for three days, with plate-bound anti-mouse-CD3 (1.5 μg/mL) and soluble anti-mouse-CD28 (1.5 μg/mL), with or without indicated cytokines and reagent. Th1 cells were induced with mouse IL-12 (10 ng/mL), and Th17 cells were induced with TGF-β1 (2 ng/mL) and mouse IL-6 (50 ng/mL). Metformin (5 mM) was used to suppress mTOR pathway. (**a**–**d**) Frequencies of IL-4^+^CD4^+^ Th2 (**a**, **b**), and IL-10^+^CD4^+^ Tr1 (**c**, **d**) cells in T cells cultured under indicated conditions. (**e** and **f**) Frequencies of IL-4^+^CD4^+^ Th2 (**e**) and IL-10^+^CD4^+^ Tr1 (**f**) cells in T cells cultured under indicated cell culture conditions. Data are representative of three independent experiments (**a** and **c**) or are pooled from three independent experiments (**b**, **d**–**f**). Summary data are presented as mean ± SD.

Figure. S10.


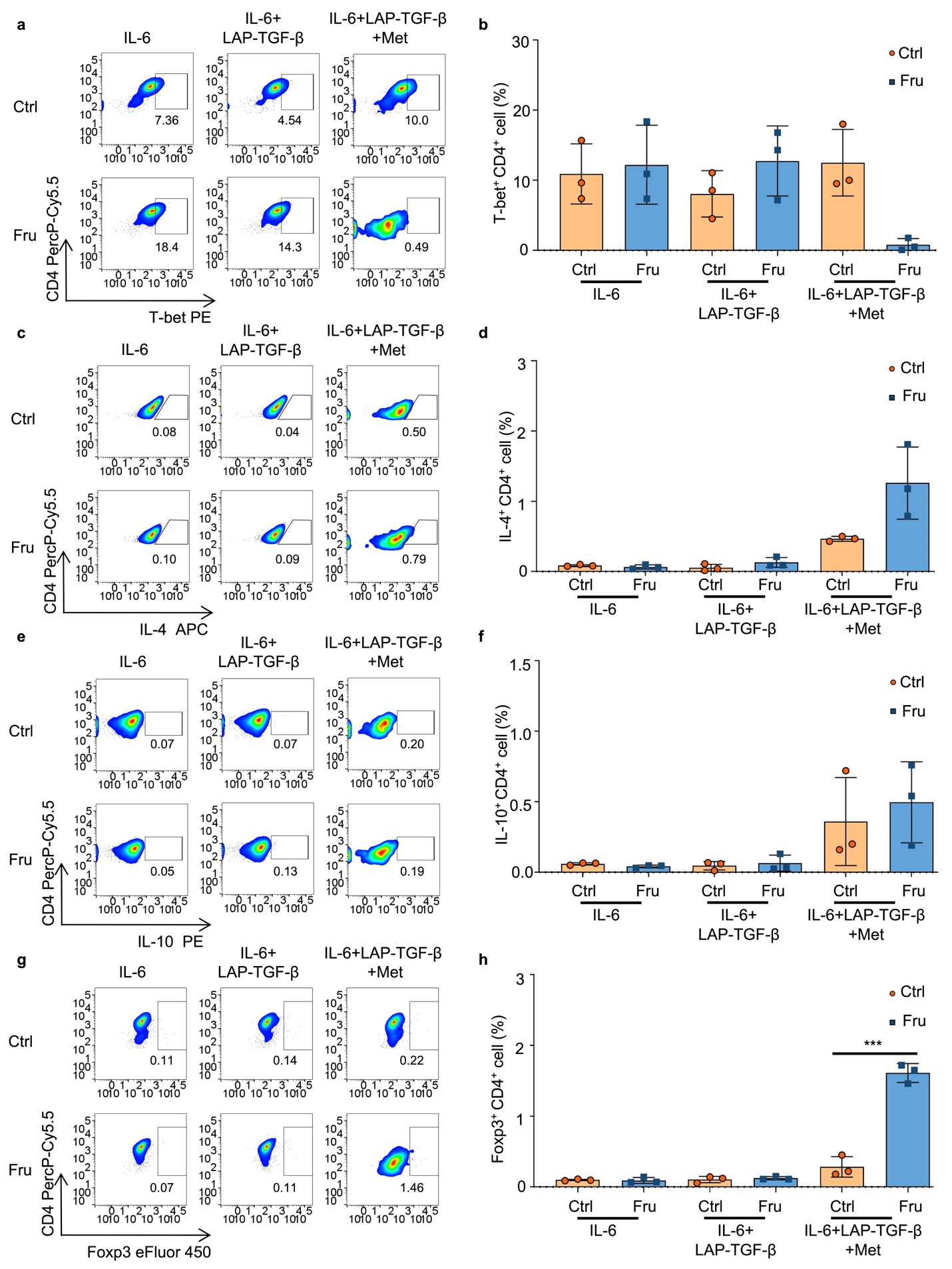


**Supplementary Fig. 10.** Metformin inhibits fructose-induced TGF-β activation by suppressing ROS production. CD4^+^CD25^-^CD62L^+^ naïve T cells were cultured in cDMEM containing 25 mM glucose or fructose for three days, with plate-bound anti-mouse-CD3 (1.5 μg/mL), soluble anti-mouse-CD28 (1.5 μg/mL), and mouse IL-6 (50 ng/mL), with or without human latent TGF-β1 (10 ng/mL) and metformin (5 mM). (**a**–**h**) Frequencies of T-bet^+^CD4^+^ T (**a**, **b**), IL-4^+^CD4^+^ Th2 (**c**, **d**), IL-10^+^CD4^+^ Tr1 (**e**, **f**), and Foxp3^+^Treg cells (**g**, **h**) in T cells cultured for three days under indicated cell culture conditions. Data are representative of three independent experiments (**a**, **c**, **e**, and **g**) or are pooled from three independent experiments (**b**, **d**, **f**, and **h**). Unpaired two-tailed Student’s t-tests were used to calculate statistical significance. Summary data are presented as mean ± SD. ***p < 0.001.

Figure. S11.


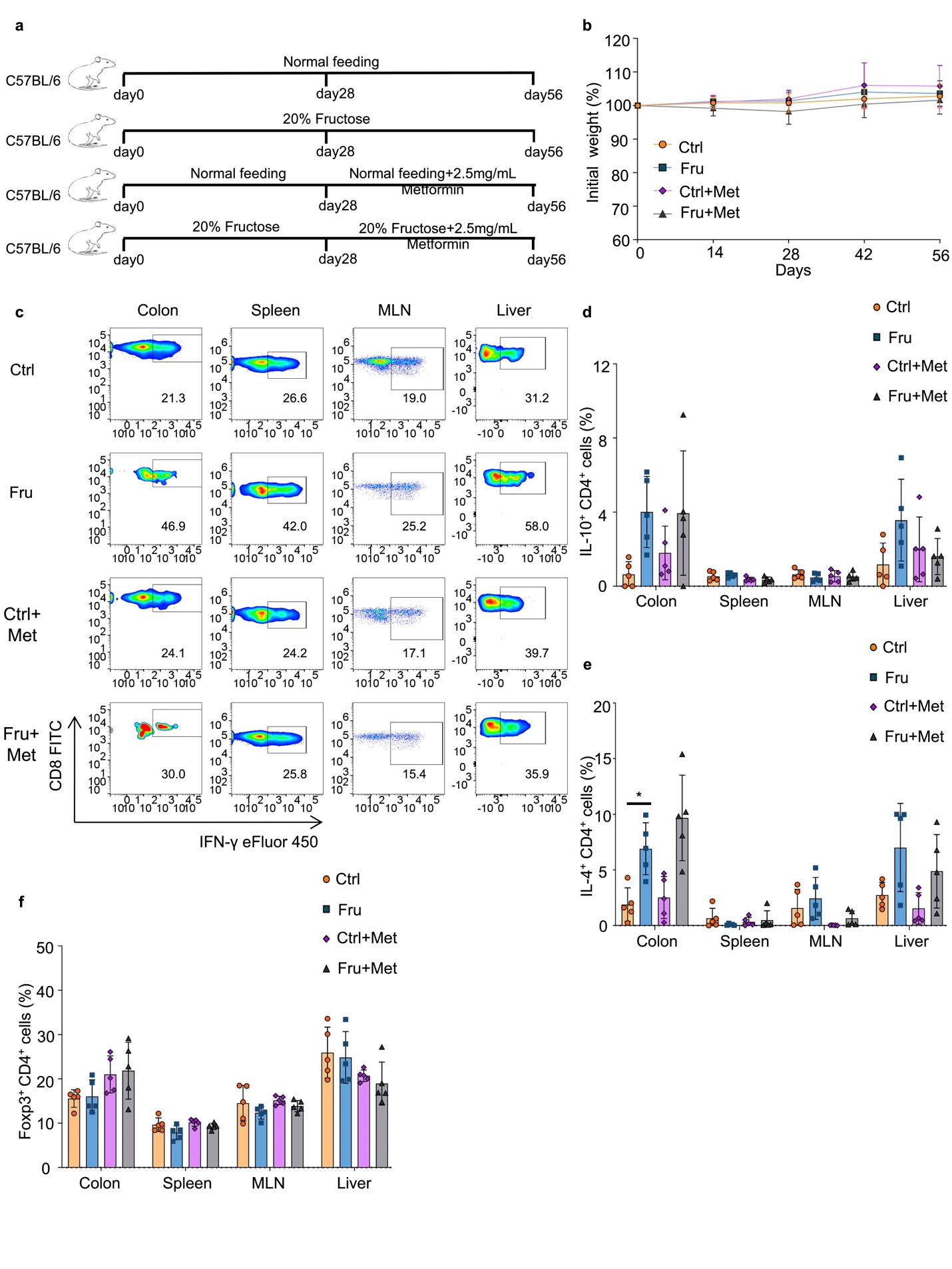


**Supplementary Fig. 11.** Metformin supplementary inhibits high fructose consumption-induced T cell inflammation *in vivo*. C57BL/6 mice were treated with regular drinking or 20% fructose water for eight weeks and half of these mice were treated with metformin (2.5 mg/mL) during the fifth to eighth weeks. T-cell immune responses were determined after the mice were euthanized (n = 5 mice per group). (**a**) Experimental scheme of the metformin supplementary experiment. (**b**) Changes in body weight during the modeling period. (**c**–**f**) Frequencies of IFN-γ^+^CD8^+^ Tc1 (**c**), IL-10^+^CD4^+^ Tr1 (**d**), IL-4^+^CD4^+^ Th2 (**e**), and Foxp3^+^ CD4^+^ Treg (**f**) cells in the colon, spleen, MLN, and liver tissues of the mice. Data are representative of two independent experiments (**b**–**f**). Unpaired two-tailed Student’s t-tests were used to calculate statistical significance. Summary data are presented as mean ± SD. *p < 0.05.

Figure. S12.


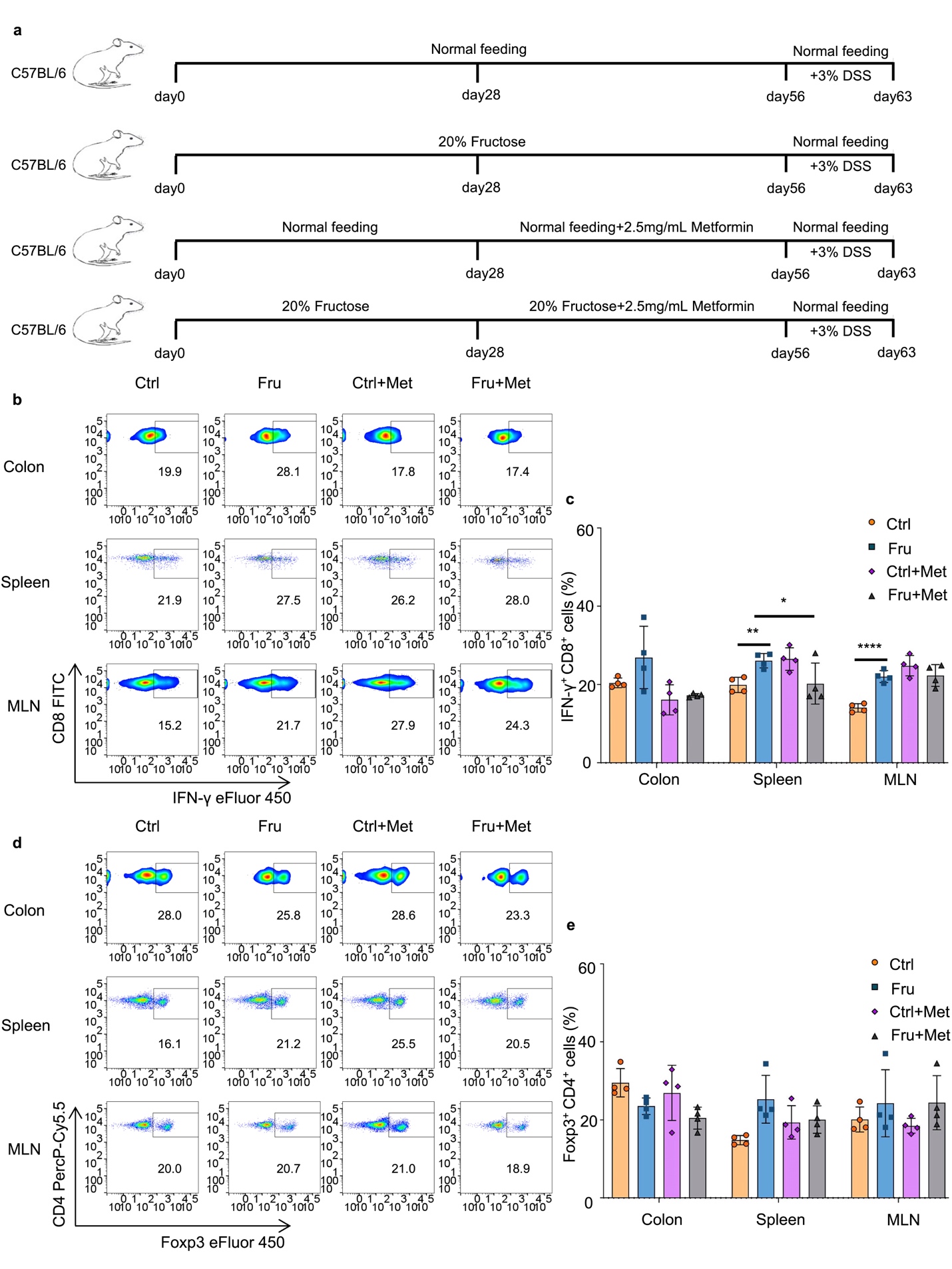


**Supplementary Fig. 12**. Metformin supplementary inhibits high fructose consumption-induced colitis aggravation. C57BL/6 mice were treated with regular drinking or 20% fructose water for eight weeks and half of these mice were treated with metformin (2.5 mg/mL) during the fifth to eighth weeks, and a DSS-induced colitis model was established to investigate the disease development after fructose water treatment (n = 4 mice per group). (**a**) Experimental scheme of the DSS-induced colitis model treated with metformin. (**b**, **c**) Frequencies of IFN-γ^+^CD8^+^ Tc1 cells in the colon, spleen, and MLN. (**d**, **e**) Frequencies of Foxp3^+^CD4^+^ Treg cells in the colon, spleen, and MLN of the mice. Data are representative of two independent experiments (**b**–**e**). One-way ANOVA (with Tukey’s multiple-comparisons post-tests) was used to calculate statistical significance. Summary data are presented as mean ± SD. *p < 0.05; **p < 0.01; ****p < 0.0001.

Figure. S13.


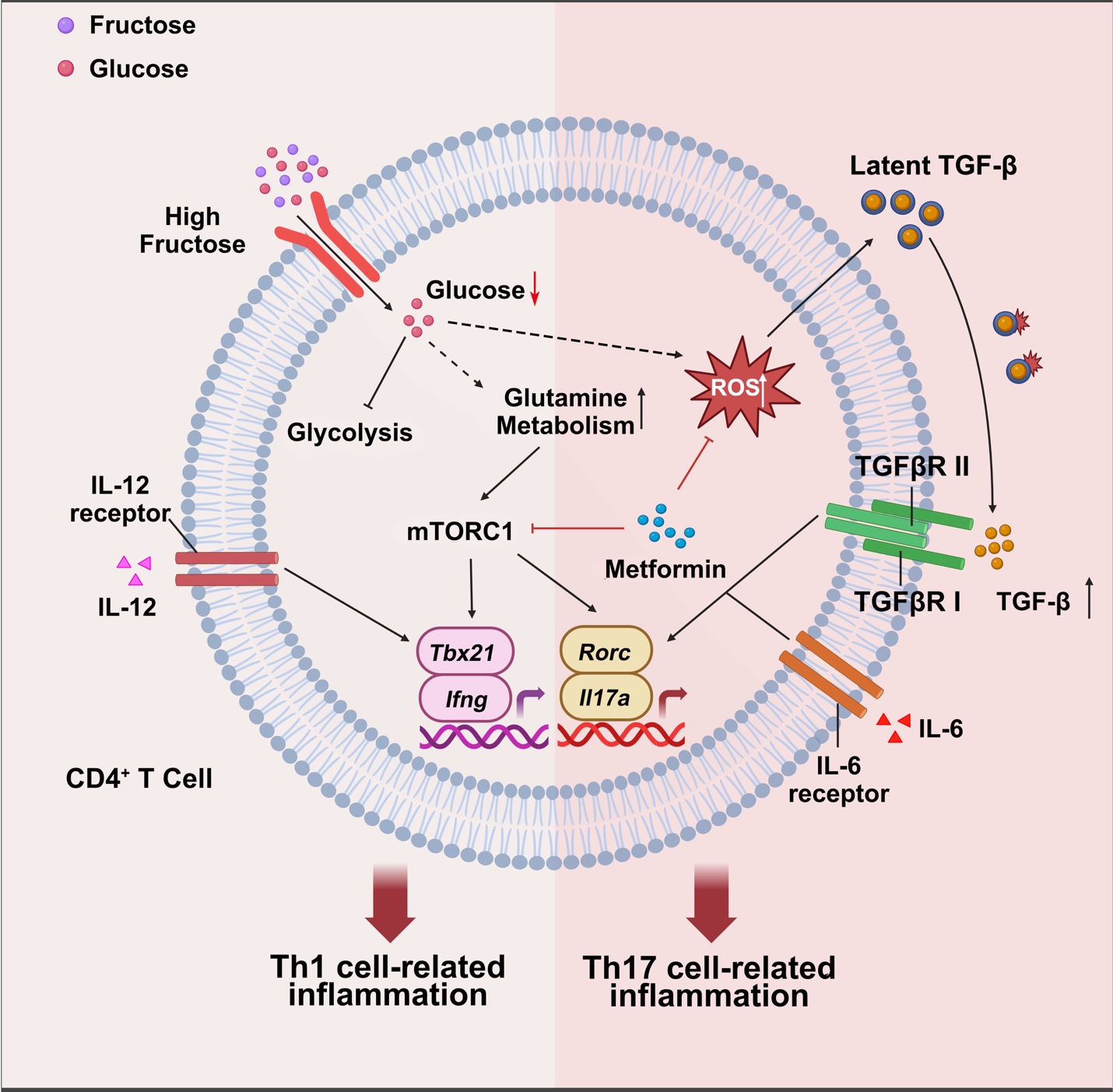


**Supplementary Fig. 13.** A proposed model for fructose-induced Th1 and Th17 cell generations in T cells. T cells do not express fructose transporter GLUT5 and cannot effectively utilize fructose. Therefore, high fructose microenvironment reduces glucose level in the internal environment and suppresses T cell glycolysis, leading to a compensatory increase in glutamine metabolism in CD4^+^ T cells. The increased glutamine metabolism induces the activation of the mechanistic target of rapamycin complex 1 (mTORC1) by up-regulating the levels of phospho-mTOR (Ser2448), resulting the differentiation of Th1 and Th17 cells. Moreover, high fructose may induce the production of reactive oxygen species (ROS) in CD4^+^ T cells, and ROS-induced transforming growth factor-β (TGF-β) activation is also involved in fructose-induced Th17 cell generation in the presence of IL-6. Besides, metformin reverses fructose-induced Th1 and Th17 cell generation by suppressing mTORC1 activation and reducing ROS-mediated TGF-β activation. This figure was created with BioRender.com.

Supplementary table 1.

The primer sequences used for Q-PCR.

|  | Q-PCR primer sequence |
| --- | --- |
| HPRT-F | TCAGTCAACGGGGGACATAAA |
| HPRT-R | GGGGCTGTACTGCTTAACCAG |
| IFNγ-F | ATGAACGCTACACACTGCATC |
| IFNγ-R | CCATCCTTTTGCCAGTTCCTC |
| T-bet-F | GATCACTCAGCTGAAAATCGAC |
| T-bet-R | AGGCTGTGAGATCATATCCTTG |
| IL17a-F | GCCAAGGGAGTTAAAGACTTTG |
| IL17a-R | TGCTACTGTTGATGTTGGGAC |
| RORγt-F | ACAAATTGAAGTGATCCCTTGC |
| RORγt-R | GGAGTAGGCCACATTACACTG |

Supplementary table 2.

Supplementary Key Resources Table.

Reagents and materials

| Reagent or Resource | source | Identifier |
| --- | --- | --- |
| Antibodies |  |  |
| Purified anti-mouse CD3 (145-2C11) | Bio X Cell | Cat# BE0001-1; RRID: AB_1107634 |
| Purified anti-mouse CD28 (37.51) | Bio X Cell | Cat# BE0015-1; RRID: AB_1107624 |
| anti-mouse/human/rat/monkey/hamster/canine/ bovine TGF-β (1D11.16.8) | Bio X Cell | Cat# BE0057; RRID: AB_1107757 |
| Anti-mouse CD4 PerCP-Cy5.5 (RM4-5) | Thermo Fisher Scientific | Cat# 45-0042-82; RRID: AB_1107001 |
| Anti-mouse CD8a FITC (53-6.7) | Thermo Fisher Scientific | Cat# 11-0081-85; RRID: AB_464916 |
| Anti-mouse CD8b FITC (H35-17.2) | Thermo Fisher Scientific | Cat# 11-0083-85; RRID: AB_657766 |
| Anti-mouse CD45.2 APC-eFluor 780 (104) | Thermo Fisher Scientific | Cat# 47-0454-82; RRID: AB_1272175 |
| Anti-mouse TCRβ APC-eFluor 780 (H57-597) | Thermo Fisher Scientific | Cat# 47-5961-82; RRID: AB_1272173 |
| Anti-mouse FOXP3 eFluor 450 (FJK-16 s) | Thermo Fisher Scientific | Cat# 48-5773-82; RRID: AB_1518812 |
| Anti-mouse RORgt APC (B2D) | Thermo Fisher Scientific | Cat# 17-6981-82; RRID: AB_2573254 |
| Anti-mouse IL-17A PE-Cy7 (eBio17B7) | Thermo Fisher Scientific | Cat# 25-7177-82; RRID: AB_10732356 |
| Anti-mouse IFN gamma eFluor 450 (XMG1.2) | Thermo Fisher Scientific | Cat# 48-7311-82; RRID: AB_1834366 |
| Anti-mouse T-bet PE (eBio4B10 (4B10)) | Thermo Fisher Scientific | Cat# 12-5825-82; RRID: AB_925761 |
| Anti-mouse IL-10 APC (JES5-16E3) | Thermo Fisher Scientific | Cat# 17-7101-82; RRID: AB_469502 |
| Anti-mouse IL-4 PE (11B11) | Thermo Fisher Scientific | Cat# 12-7041-82; RRID: AB_395391 |
| Anti-mouse IL-10 PE (JES5-16E3) | Thermo Fisher Scientific | Cat# 12-7101-82; RRID: AB_466176 |
| Anti-mouse IL-4 PE-Cy7 (11B11) | Thermo Fisher Scientific | Cat# 25-7041-82; RRID: AB_2573520 |
| Anti-mouse IL-4 APC (11B11) | Thermo Fisher Scientific | Cat# 17-7041-82; RRID: AB_469494 |
| Anti-mouse CD62L (L-Selectin) FITC (MEL-14) | Thermo Fisher Scientific | Cat# 11-0621-85; RRID: AB_465110 |
| Anti-mouse CD69 PE (H1.2F3) | Thermo Fisher Scientific | Cat# 11-0621-85; RRID: AB_465110 |
| Anti-mouse CD44 APC (IM7) | Thermo Fisher Scientific | Cat# 17-0441-83; RRID: AB_469391 |
| Anti-mouse CD25 PE-Cyanine7 (PC61.5) | Thermo Fisher Scientific | Cat# 25-0251-82; RRID: AB_469608 |
| β-Actin (13E5) Rabbit mAb | CST | Cat# 4970; RRID: AB_2223172 |
| mTOR (7C10) Rabbit mAb | CST | Cat# 2983; RRID: AB_2105622 |
| Phospho-mTOR (Ser2448) (D9C2) XP® Rabbit mAb | CST | Cat# 5536; RRID: AB_10693423 |
| Chemicals, Peptides, and Recombinant Proteins |  |  |
| Recombinant Human Latent TGF-β1 | R&D Systems | Cat# 299-LT-005 |
| Recombinant mouse IL-6 | PEPROTECH | Cat# 406-ML-200 |
| Recombinant Human TGF-β1 | PEPROTECH | Cat# 100-21C-50UG |
| Recombinant Murine IL-12 p70 | PEPROTECH | Cat# 200-12 |
| Glutaminase inhibitors ,CB839 | selleck | Cat# 1439399-58-2 |
| Rapamycin | selleck | Cat# S1039 |
| Metformin HCl | selleck | Cat# S1950 |
| Metformin HCl | Macklin | Cat# 1115-70-4 |
| Dextran Sulfate Sodium Salt, DSS | YEASEN | Cat# 9011-18-1 |
| SB431542 | Selleck.cn | Cat# S1067 |
| N-Acetyl-L-cysteine (NAC) | Medchemexpress | Cat# HY-B0215 |
| D-(+)-Glucose | Sigma | Cat# G6152-500G |
| D-(-)-Fructose | Sigma | Cat# F0127-500G |
| D-Fructose | Macklin | Cat# D809612 |
| DNase I | Aladdin | Cat# D106200-1g |
| Collagenase IV | worthington | Cat# WBC-LS004189 |
| Liberase TL | Roche | Cat# 05401020001 |
| PMA | Sigma | Cat# P8139 |
| Ionomycin calcium salt | Aladdin | Cat# I133497-25mg |
| Golgi-Plug Protein Transport Inhibitor | BD Biosciences | Cat# 555029 |
| rh Annexin V Pacific Blue™ | Thermo Fisher Scientific | Cat# A35122 |
| 7-AAD Viability Staining Solution | Thermo Fisher Scientific | Cat# 00-6993-50 |
| Annexin V Binding Buffer | Thermo Fisher Scientific | Cat# V13246 |
| Zombie Yellow Fixable Viability Kit | BioLegend | Cat# 423104 |
| EDTA | Solarbio | Cat# E1170 |
| DTT (dithiothreitol) | Thermo Fisher Scientific | Cat# R0862 |
| Corning Cell-Tak Cell and Tissue Adhesive | Life Sciences | Cat# 354240 |
| XF RPMI Medium, PH 7.4 | Agilent | Cat# 103576-100 |
| Glucose free DMEM | Thermo Fisher Scientific | Cat# 11966025 |
| Opti-MEM™ | Thermo Fisher Scientific | Cat# 31985070 |
| Fetal Bovine Serum (FBS) | Vivaccell | Cat# C04001-500 |
| Penicillin-Streptomycin | Thermo Fisher Scientific | Cat# 15140122 |
| L-Glutamine | Thermo Fisher Scientific | Cat# 25030081 |
| Sodium Pyruvate | Thermo Fisher Scientific | Cat# 11360070 |
| MEM Non-Essential Amino Acids Solution | Thermo Fisher Scientific | Cat# 11360070 |
| 2-Mercaptoethanol | Sigma | Cat# M3148 |
| HEPES Buffer | Thermo Fisher Scientific | Cat# 118-089-721 |
| Bovine Serum Albumin (BSA) | Sigma | Cat# B2064 |
| Bovine Serum Albumin (BSA) | BioFroxx | Cat# 4240GR100 |
| ACK Lysing buffer | Thermo Fisher Scientific | Cat# A1049201 |
| Critical Commercial Assays |  |  |
| CD4^+^ CD62L^+^ T cell Isolation Kit, mouse | Miltenyi Biotec | Cat# 130-106-643 |
| CD4^+^ T Cell Isolation Kit, mouse | Miltenyi Biotec | Cat# 130-095-248 |
| Foxp3/Transcription Factor Staining Buffer Set | Thermo Fisher Scientific | Cat# 00-5523-00 |
| Cytofix/Cytoperm Fixation/ PermeabilizationSolution Kit | BD Biosciences | Cat# 554714 |
| DCFDA / H2DCFDA - Cellular ROS Assay Kit | Medchemexpress | Cat# HY-D0940 |
| CellTrace CFSE Cell Proliferation Kit | Thermo Fisher Scientific | Cat# C34554 |
| Total RNA rapid extraction kit | JIANSHI BIOTECH | Cat# TR205-200 |
| PrimeScript™ RT reagent Kit with gDNA Eraser (Perfect Real Time) | Takara | Cat# RR047A |
| TB Green® Premix Ex Taq™ II (Tli RNaseH Plus) | Takara | Cat# RR820A |
| BCA protein concentration determination kit | Solarbio | Cat# PC0020 |
| SDS-PAGE Gel Kit | CWBIO | Cat# CW0022S |
| Hypersensitive ECL chemiluminescence kit | Beyotime | Cat# P00185 |
| Seahorse XF Cell Mito Stress Test Kit | Agilent | Cat# 103015-100 |
| Full Protein Extraction Kit (strong) | Solarbio | Cat# BC3710 |
| ProteanFect CRISPRMax Cas9 Mouse Primary Immune Cell Gene Editing Transfection Kit | Nanoportal Biotech | Cat# PT06 |
| Fructose content test kit | Suzhou Grace Biotechnolgy Co.,Ltd | Cat# G0530W |
| Experimental Models: Organisms/Strains |  |  |
| Mouse: C57BL/6 | Jackson Lab | Cat# 000664; RRID: IMSR_JAX:000664 |
| Mouse: Rag1^-/-^ | Jackson Lab | Cat# 002216; RRID:IMSR_JAX:002216 |
| Mouse: BALB/c-nu | Huafukang Biotechnology Co., LTD | Cat# NO.SM-014;RRID: IMSR_RJ: BALB-C-NUDE |
| Software and Algorithms |  |  |
| FlowJo 9 or FlowJo 10 software | FlowJo | https://www.flowjo.com/solutions/flowjo  RRID: SCR_008520 |
| GraphPad Prism 7 software | GraphPad Software | https://www.graphpad.com/  RRID: SCR_002798 |
| Other |  |  |
| BD LSRFortessa | BD Biosciences | N/A |
| Real-time fluorescence quantitative PCR instrument | Analytik Jena | qTOWER3G |
| Seahorse XFe96 Analyzers | Agilent | N/A |

Original western blots. (separate file)

Gating strategies for the flow cytometry data. (separate file)
