# Supplementary material for: High fructose consumption aggravates inflammation by promoting effector T cell generation via inducing metabolic reprogramming
Source: Signal Transduct Target Ther. 2025 Aug 26;10:271. doi: 10.1038/s41392-025-02359-9 (PMC12379281; doi:10.1038/s41392-025-02359-9)
Supplement: Supplementary file 2 — Supplementary_Materials_2 [file 41392_2025_2359_MOESM2_ESM.pptx]

## Slide 1
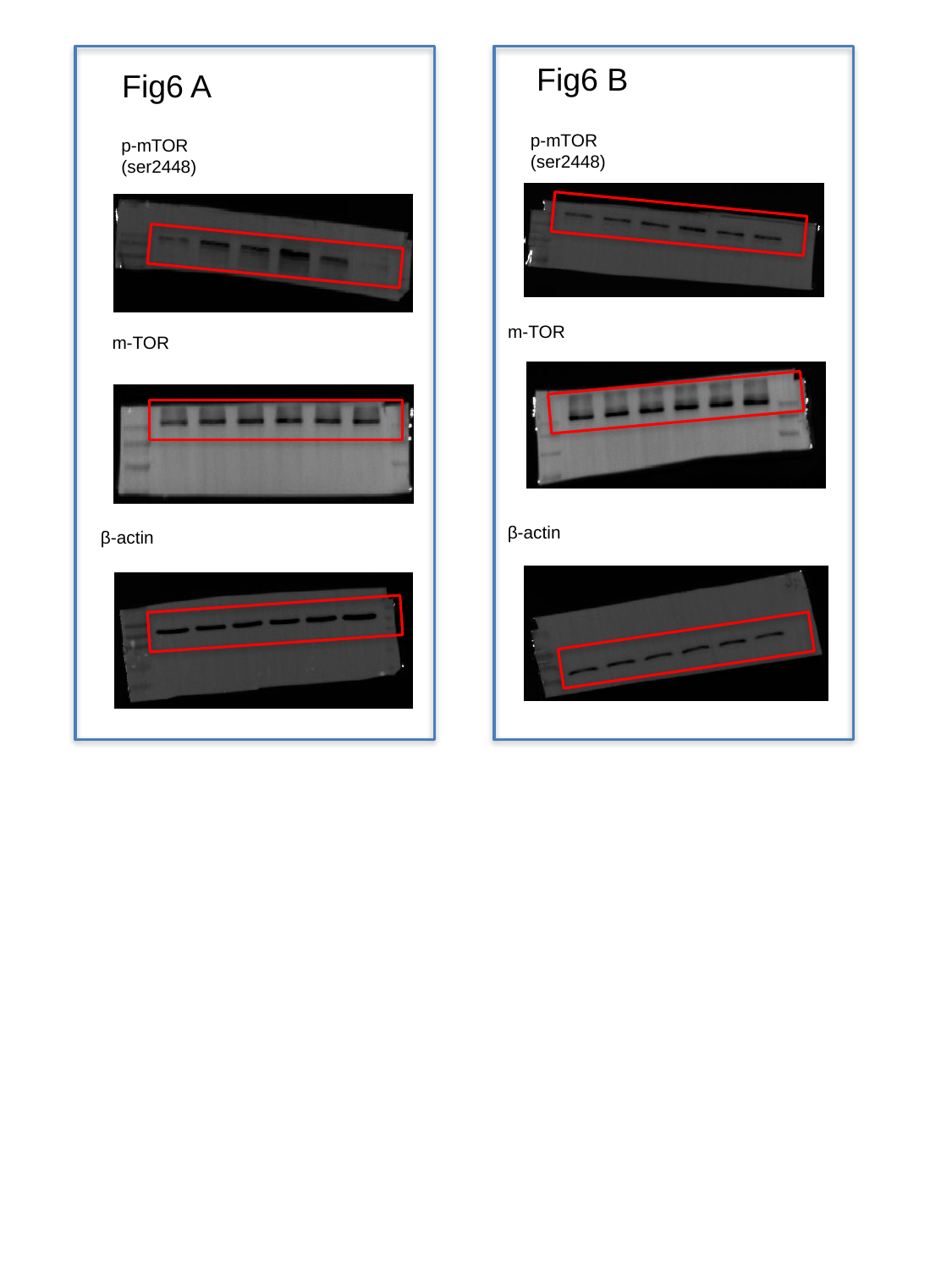

Fig6 A
p-mTOR
(ser2448)
m-TOR
β-actin
Fig6 B
p-mTOR
(ser2448)
m-TOR
β-actin

## Slide 2
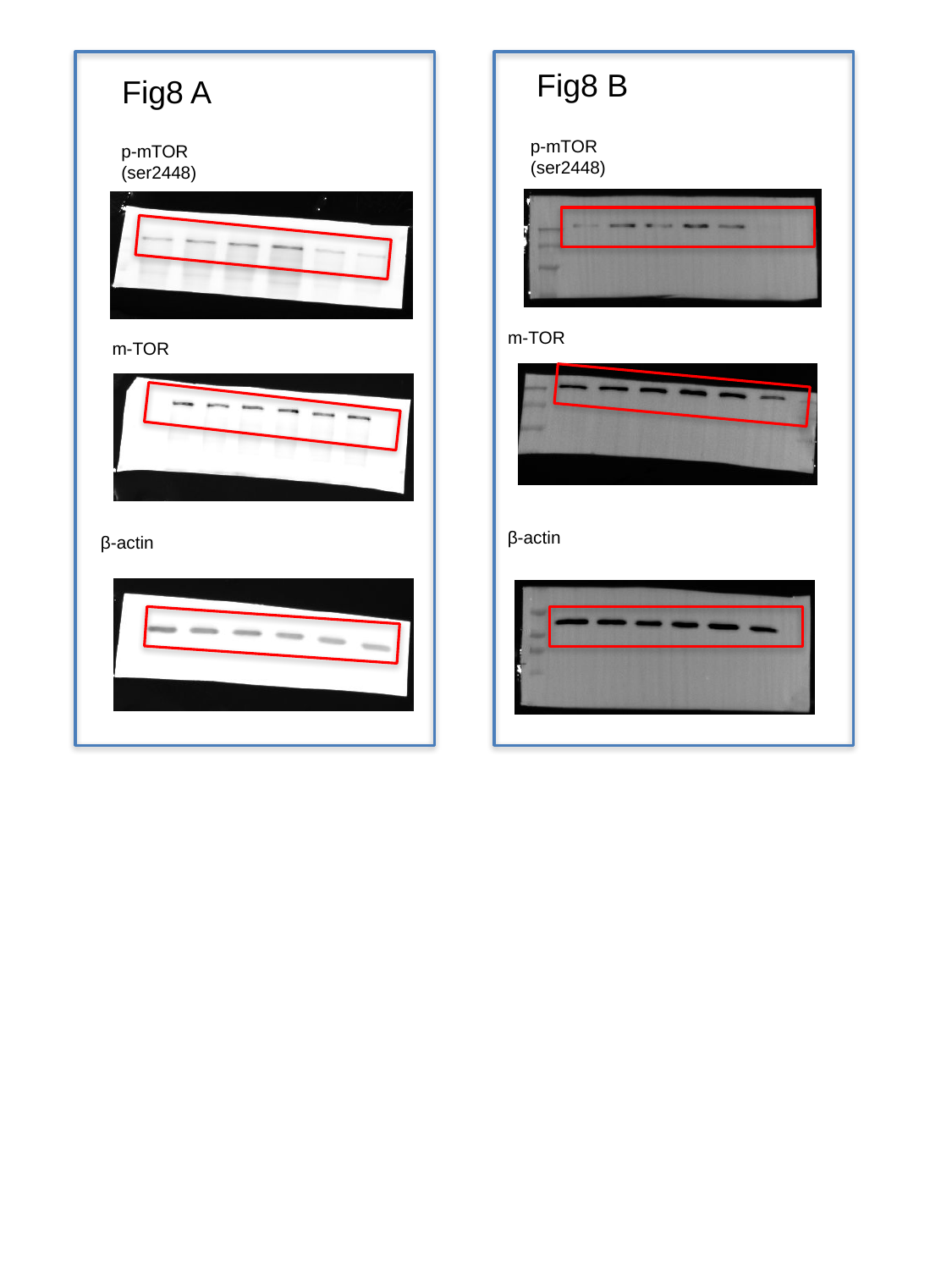

Fig8 A
p-mTOR
(ser2448)
m-TOR
β-actin
Fig8 B
p-mTOR
(ser2448)
m-TOR
β-actin
