# Supplementary material for: High fructose consumption aggravates inflammation by promoting effector T cell generation via inducing metabolic reprogramming
Source: Signal Transduct Target Ther. 2025 Aug 26;10:271. doi: 10.1038/s41392-025-02359-9 (PMC12379281; doi:10.1038/s41392-025-02359-9)
Supplement: Supplementary file 3 — Supplementary_Materials_3 [file 41392_2025_2359_MOESM3_ESM.pptx]

## Slide 1
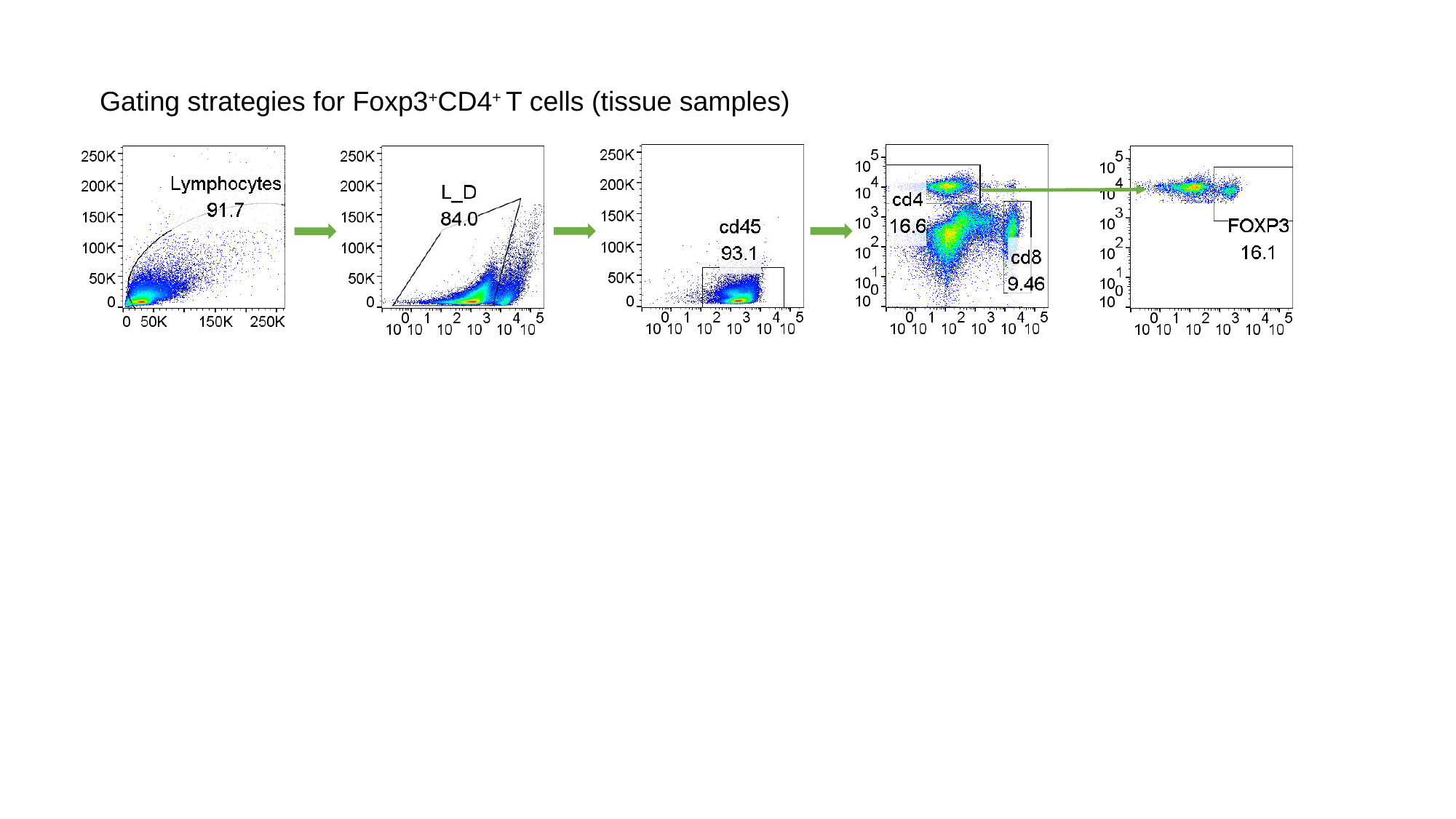

Gating strategies for Foxp3+CD4+ T cells (tissue samples)

## Slide 2
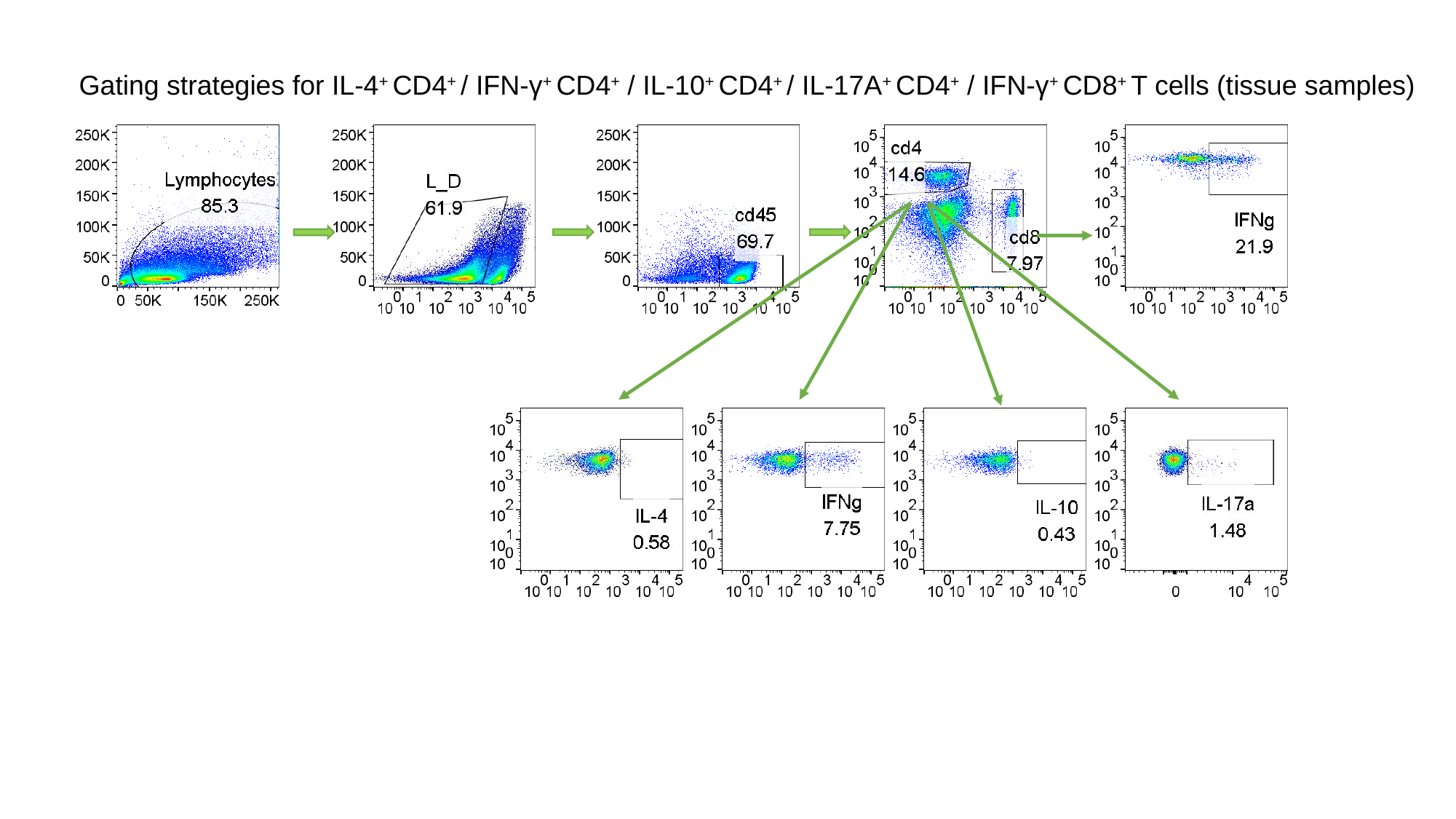

Gating strategies for IL-4+ CD4+ / IFN-γ+ CD4+ / IL-10+ CD4+ / IL-17A+ CD4+ / IFN-γ+ CD8+ T cells (tissue samples)

## Slide 3
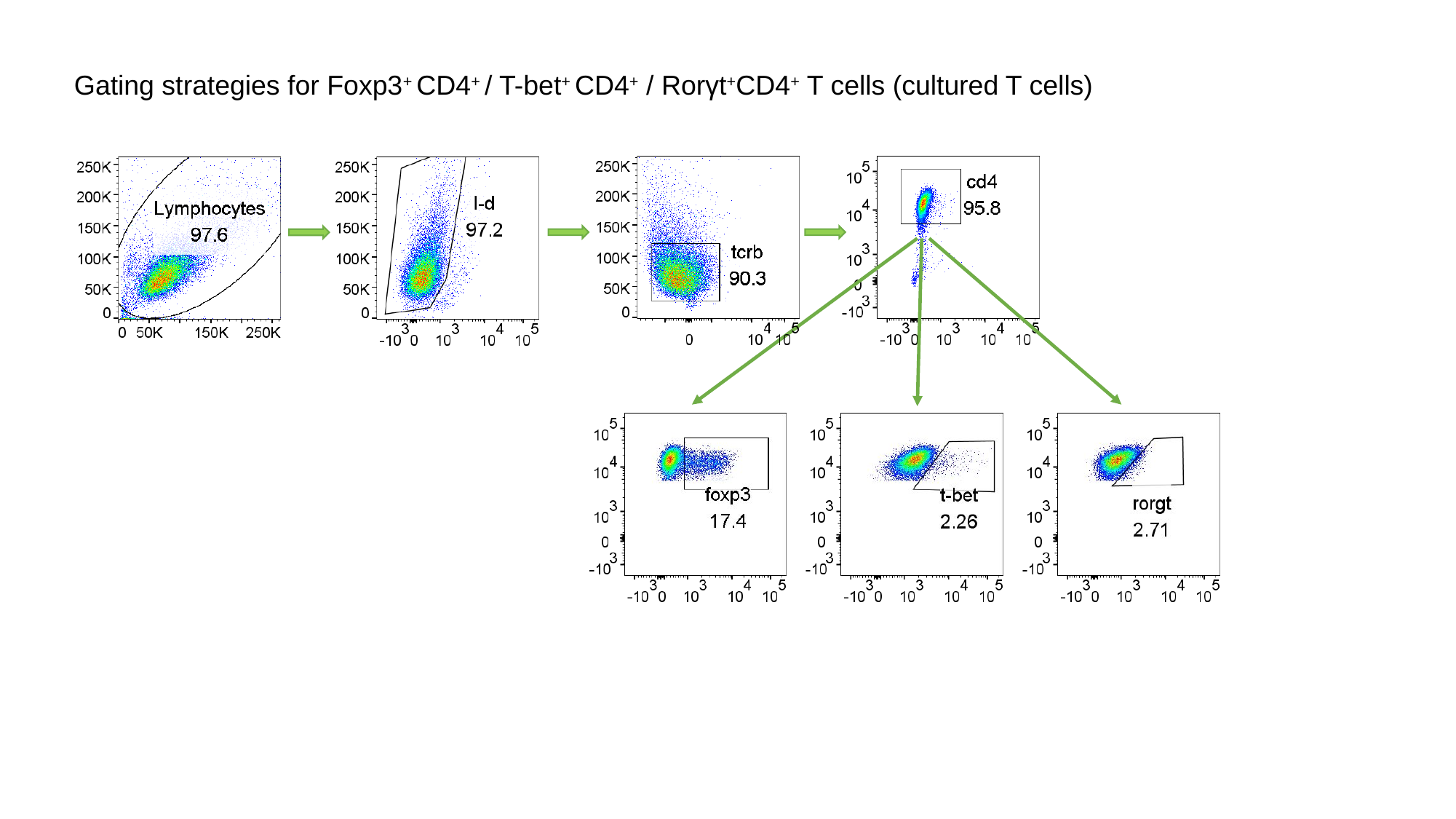

Gating strategies for Foxp3+ CD4+ / T-bet+ CD4+ / Rorγt+CD4+ T cells (cultured T cells)

## Slide 4
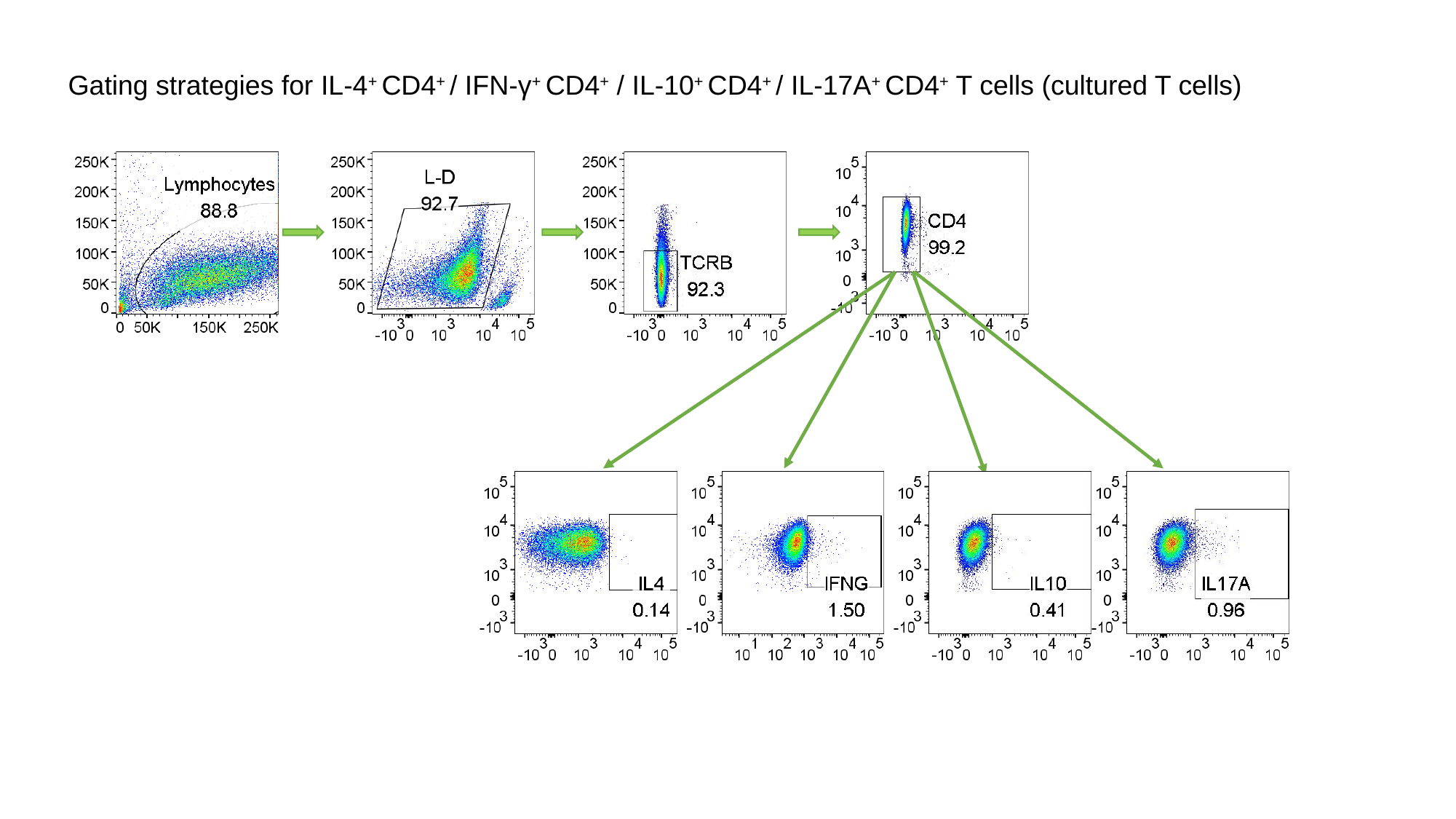

Gating strategies for IL-4+ CD4+ / IFN-γ+ CD4+ / IL-10+ CD4+ / IL-17A+ CD4+ T cells (cultured T cells)
